# MammoDiff: weakly supervised mammographic anomaly detection via classifier-guided denoising diffusion models—a real-world clinical study on the KHCC mammography cohort

**DOI:** 10.3389/fmedt.2026.1912196

**Published:** 2026-10-12

**Authors:** Ahmad Nasayreh, Nagham Osman, Laila Tahoun, Asem Mansour, Iyad Sultan

**Affiliations:** 1 Department of Computer Engineering, Automation and Robotics, School of Computer and Telecommunication Engineering, University of Granada, Granada, Spain; 2 Artificial Intelligence Office, King Hussein Cancer Center, Amman, Jordan; 3 Department of Electronics and Electrical Engineering, UCL, London, United Kingdom; 4 AI Center, UCL, London, United Kingdom; 5 Department of Radiology, King Hussein Cancer Center, Amman, Jordan; 6 Department of Pediatrics, King Hussein Cancer Center, Amman, Jordan

**Keywords:** anomaly detection, diffusion model, generative models, mammography, weakly supervised

## Abstract

**Introduction:**

Early detection of malignant mammographic lesions is a critical clinical challenge, particularly where large annotated datasets are unavailable. Supervised deep learning achieves near-radiologist performance but requires large datasets with reliable labels and pixel/ROI annotations. Weakly supervised anomaly detection, requiring only image-level binary labels, offers an annotation-efficient alternative, yet prior GAN- and autoencoder-based methods have struggled to preserve fine anatomical detail in the mammographic domain.

**Methods:**

We present MammoDiff, to our knowledge the first application of classifier-guided denoising diffusion models to weakly supervised mammographic anomaly detection, trained and evaluated on 3,013 mammograms from 806 patients at King Hussein Cancer Center (KHCC), Jordan. MammoDiff encodes input anatomy through deterministic DDIM noise encoding and reconstructs a counterfactual healthy image guided by the gradient signal of a noise-conditioned binary classifier. The anomaly map is the pixel-wise absolute difference between input and reconstruction, and the image-level score is obtained by spatial mean pooling. A 3 × 3 ablation over noise level *L* ∈ {250, 500, 750} and gradient scale *s* ∈ {50, 100, 200} was performed on the validation set.

**Results:**

The optimal configuration (*L* = 750, *s* = 50) achieved AUROC = 0.7246 [95% CI: 0.6685, 0.7825] on the held-out test set (*n* = 304), compared with a simplified PatchCore-style baseline (AUROC = 0.6634) and a supervised ResNet-50 reference (AUROC = 0.8100), without pixel-level annotation. The proposed Healthy-Shift Index (HSI) was positive for 94.8% of malignant and 87.4% of normal cases, so it is the separation between the class distributions rather than the sign of the index that carries information.

**Discussion:**

This performance is not clinically deployable: at the Youden-optimal threshold the sensitivity is 0.6013, meaning that 61 of 153 malignant cases are missed, well below the approximately 87% targeted by FDA-cleared triage devices, and the study is single-centre without external validation. MammoDiff is therefore presented as a methodological proof-of-concept and a reproducible baseline for future work, not as a screening or triage tool..

## 1 Introduction

Breast cancer is the most commonly diagnosed cancer among women worldwide, and 2.3 million new cases and 685,000 deaths are registered in 2020 alone (1). Mammography is the only imaging modality proven to reduce breast cancer mortality in large, randomized trials and remains the reference standard for population screening (2, 3). However, mammogram interpretation is a perceptual task that is cognitively challenging; sensitivity of readers in clinical practice varies widely (70%–87%), and it is marked by the subtlety of lesions, breast tissue density, reader fatigue, and the volume of cases (4). These human aspects create diagnostic inconsistency, which has inspired decades of computer-assisted detection studies.

The introduction of deep learning has transformed automated mammographic analysis, with architectures such as ResNet (5), DenseNet (6), and Vision Transformers (7) achieving radiologist-competitive results on benchmark datasets. For instance (8), showed that a model trained on 76,000 mammograms could achieve an AUC of 0.886, outperforming human experts. However, the transition from successful benchmarks to clinical practice is bottlenecked by the heavy reliance on high-quality, expertly labeled data. Such datasets are inherently difficult to obtain, creating a barrier for clinical centers in resource-constrained settings, such as the Middle East, where the infrastructure for large-scale data curation may not yet be fully established.

Weakly supervised anomaly detection offers a compelling alternative: models require only coarse image-level binary labels (healthy vs. diseased), entirely circumventing the need for pixel-wise lesion annotation. Related anomaly detection approaches, spanning weakly supervised, one-class, and unsupervised paradigms include autoencoders (9), variational autoencoders (10), normalising flows (11), and generative adversarial networks (12, 13). These algorithms reassemble input images under a healthy-distribution prior, and the reconstruction difference is the anomaly signal. In mammography specifically, this paradigm has been used to flag dense-tissue regions whose reconstruction error exceeds that of surrounding parenchyma; however, such methods have historically struggled with subtle microcalcification clusters and ill-defined mass borders, both of which produce only low-magnitude reconstruction differences relative to the noisy glandular background; comparable difficulties have been reported for semi-supervised anomaly segmentation in other medical imaging domains (14). But GAN-based algorithms are infamously prone to training instabilities and mode collapses (15), whereas autoencoder-based methods often trade off fine-grained structural detail, which is clinically important, especially with mammographic microcalcifications and spiculated masses.

The new generative paradigm, Denoising Diffusion Probabilistic Models (DDPMs) (16), has been found to overcome both of these limitations, with better image fidelity, training stability, and sample diversity compared to GANs (28). Introduced a seminal diffusion-based anomaly detection framework (18) presented the combination of the deterministic encoding properties of Denoising Diffusion Implicit Models (DDIMs) (19) with classifier guidance (28) to transform diseased images into the healthy class distribution. A key advantage of this framework is that it maintains fine anatomical structure with lossless reversed-DDIM encoding, followed by steering the denoising trajectory by the gradient of a noise-conditioned classifier, resulting in highly localized, detail-preserving anomaly maps. Brain MRI and chest radiography validation showed up to 0.98 and 0.87 values of the AUROC on BRATS2020 and CheXpert datasets, respectively, which is significantly better than GAN and autoencoder baselines.

However, the applicability of diffusion-based anomaly detection to digital mammography, with its characteristically high-frequency texture, substantial inter-patient density variability, bilateral tissue asymmetry, and diagnostically critical microcalcification patterns, has not been systematically evaluated. Moreover, to our knowledge, no diffusion anomaly detection study has been conducted on a dataset from the Arab world, where epidemiological characteristics, breast tissue density distributions, and imaging protocol standards differ meaningfully from Western cohorts (20).

The principal contributions of this work are:

- Within the scope of the structured literature search reported in Section 2.9, we present what is to our knowledge the first application of classifier-guided denoising diffusion models to weakly supervised mammographic anomaly detection, validated on a real-world clinical dataset from King Hussein Cancer Center (KHCC), Jordan. We emphasise that the individual methodological components, namely DDIM encoding, classifier guidance and counterfactual healthy reconstruction, are all established techniques. The contribution of the present work lies in the domain transfer to mammography, in the domain-specific hyperparameter characterisation, and in the proposed HSI metric, rather than in any new diffusion architecture.
- We introduce the Healthy-Shift Index (HSI), a novel semantic interpretability metric that quantifies the directional probability shift of pathological cases toward the healthy class manifold, orthogonal to pixel-level anomaly scores, and confirm *p*(HSI > 0) = 94.8% across malignant test cases.
- We provide an honest empirical evaluation of MammoDiff as a proof-of-concept framework: AUROC = 0.7246 exceeds the simplified PatchCore-style baseline (0.6634) by 6.1 percentage points, while remaining below the supervised ResNet-50 reference (0.8100), establishing a reproducible baseline for future diffusion-based mammographic anomaly detection research.

A literature review is presented in Section 2, followed by a description of the MammoDiff approach in Section 3, which comprises the architecture and the proposed Healthy-Shift Index. Results and visualizations of the experiments and discussion of limitations are shown in Section 4, Finally, section 5 conclude the study.

## 2 Related work

Weakly supervised and unsupervised anomaly detection in medical imaging is an intersection of generative modeling, clinical decision support and low-resource medical AI. The recent generative models (VAEs, GANs, diffusion) have enhanced fidelity and stability of training, but it is still difficult to obtain reliable qualitative anomaly maps, particularly for mammography. The subsections below provide a literature review of these methodological families, with a specific focus on the contributions and limitations that are the most specific to the mammographic anomaly detection problem that is the focus of this work. Tables 1a, 1b are presented in a structured comparison format, providing a comparative overview of the fifteen closely related studies, and clearly stating the research gaps that drive MammoDiff.

**Table 1a:** Medical anomaly detection methods.

Reference	Dataset	Method family	Key contribution	Results (AUROC)	Gap/limitation
Zimmerer et al. (9)	BraTS, MOOD	Variational AE	ceVAE: latent-space anomaly scoring via ELBO	0.72 (BraTS)	No class supervision; blurry reconstructions
Chen and Konukoglu (10)	BraTS	Adversarial AE	Constrained AE for healthy-distribution modelling	0.80 (BraTS)	Limited spatial resolution of anomaly maps
Baur et al. (24)	BraTS	Multi-AE Benchmark	7 AE architectures benchmarked	0.74 (best AE)	Confirms AE performance ceiling
Baumgartner et al. (12)	CheXpert	Wasserstein GAN	Feature attribution for disease localisation	0.84 (CheXpert)	GAN instability; no deterministic encoding
Siddiquee et al. (13)	CheXpert	Fixed-Point GAN	Identity constraint for same-domain translation	0.87 (CheXpert)	Cannot preserve fine structural details
Wolleb et al. (25)	Multi-domain	DescarGAN + Attn	Disease-specific attention + cycle-GAN	0.84 (CheXpert)	GAN limitations; no mammography validation
Wolleb et al. (18)	BraTS, CheXpert	DDIM + Cls. Guidance	First diffusion-based medical anomaly detection	0.98 (BraTS)	Brain/chest only; no mammography validation
Wyatt et al. (31)	BraTS	AnoDDPM	Simplex noise for unsupervised diffusion AD	0.79 (BraTS)	No classifier guidance; lower AUROC
Roth et al. (27)	MVTec, Medical	PatchCore	Coreset memory bank + NN anomaly scoring	0.99 (MVTec)	Unsupervised (trained on normal data only); no class conditioning
Pinaya et al. (33)	UK Biobank (100k)	LDM + Transformer	Large-scale brain AD via latent diffusion	0.89 (UKB)	Requires massive dataset; high compute cost

**Table 1b:** Mammography deep learning methods.

Reference	Dataset	Supervision level	Key contribution	Results (AUC)	Gap/limitation
Kim et al. (34)	GURO (170k images)	Supervised	Evaluation of AI in a multireader setup to reduce false positives	0.959	Retrospective study; performance may vary in diverse ethnic populations
Shen et al. (21)	DDSM & INbreast	End-to-End Supervised	Whole-image classification without manual ROI annotations	0.88	Performance drops on screening-only datasets compared to diagnostic sets
Wu et al. (23)	NYU (1M images)	Supervised (Multi-view)	Massive-scale training using multi-view (CC/MLO) for radiologist-level AI	0.895	Requires enormous datasets (1M+); high computational demand
Sun et al. (35)	DDSM/INbreast	Multi-view/multi-level	Integration of multi-view features and BI-RADS categorization logic	0.93	Complex multi-level architecture; relies on high-resolution ROI patches
Schaffter et al. (36)	DREAM Challenge	External Validation	Rigorous external testing of commercial AI against independent datasets	0.85–0.92	Highlights a performance gap between internal validation and external testing
McKinney et al. (8)	UK + US (76k)	Fully Supervised	Large-scale study comparing AI vs. radiologists	0.886	Requires massive (76k) annotated datasets
Nguyen et al. (22)	VinDr-Mammo 5k	Supervised (Exam-level)	ViT for dense-tissue classification	0.91	Uses exam-level labels; requires large-scale training

### 2.1 Deep learning for mammographic analysis

The classification of mammograms using supervised deep learning has come a long way in the last decade. Shen et al. (21) showed that end-to-end trained CNNs on CBIS-DDSM can reach AUC of 0.88 for the malignancy classification task without hand-crafted features. In a landmark international multi-reader study, McKinney et al. (8) demonstrated that the deep learning system, trained with 76,250 mammograms, performed better than six certified radiologists, with a 5.7% decrease in false positives and a 9.4% decrease in false negatives, making AI a clinically viable screening adjunct. Recently, Nguyen et al. (22) published the large-scale Vietnamese benchmark VinDr-Mammo consisting of 5,000 full-field digital mammograms with BI-RADS annotations, and reported an AUC of 0.91 for a Vision Transformer (ViT) based classifier on dense tissue subtypes. More recently, Wu et al. (23) proposed a deep neural network for multi-view mammography that jointly models craniocaudal and mediolateral oblique projections, achieving AUC of 0.876 on NYU-Mammo. Many supervised approaches require large datasets with reliable exam-level labels, and localization/interpretability often benefits from box/pixel annotations, which are expensive to curate.

### 2.2 Autoencoder-based weakly supervised anomaly detection

Zimmerer et al. (9) introduced the context-encoding variational autoencoder (ceVAE), combining VAE reconstruction error with density estimation in latent space. Applied to brain MRI, this approach achieved AUROC of 0.72 on BraTS, establishing the core principle of healthy-distribution modelling for anomaly scoring. Chen and Konukoglu (10) extended this with constrained adversarial autoencoders that enforce latent space regularisation through discriminator feedback, demonstrating improved anomaly localisation in brain lesion detection with AUC improvements of 8% over standard VAEs. Baur et al. (24) conducted a systematic benchmark of seven autoencoder architectures, VAE, AAE, VQVAE, f-AnoGAN, ceVAE, KL-VAE, and conditional VAE, for unsupervised brain MRI anomaly detection, finding that no single architecture consistently outperformed others and that reconstruction quality did not reliably predict anomaly sensitivity. This benchmark motivated the exploration of fundamentally different generative paradigms. In mammography specifically, the diagnostic signal is carried largely by microcalcifications and other high-frequency textures, which is precisely the information that reconstruction-based methods preserve least well.

### 2.3 GAN-based anomaly detection and image translation

Generative Adversarial Networks (15) provided an early framework for weakly supervised disease localisation through image-to-image translation. Baumgartner et al. (12) leveraged Wasserstein GANs with gradient penalty and visual feature attribution to generate counterfactual healthy images, producing coarse-grained disease attribution maps. Siddiquee et al. (13) proposed Fixed-Point GAN (FP-GAN), which enforces an identity constraint for same-domain inputs, ensuring that healthy input images are reconstructed without modification while diseased inputs undergo targeted translation, yielding sharper anomaly maps than unconstrained image translation. Wolleb et al. (25) introduced DescarGAN, combining disease-specific attention mechanisms with weakly supervised cycle-consistent adversarial training, and demonstrated that explicit attention gates substantially improved spatial specificity of anomaly maps on chest radiography and brain MRI. Despite these contributions, GAN-based methods suffer from well-documented training instability, mode collapse, sensitivity to hyperparameters (15), and can produce anatomically implausible edits, motivating the exploration of diffusion-based alternatives.

### 2.4 Normalizing flows and memory-based methods

The normalizing flows-based architecture (DifferNet) by Rudolph et al. (26) was suggested as a method of anomaly detection in industry and medicine was designed to model the normal feature distribution through exact likelihood estimation on top of CNN-extracted features. Though conceptually sound, normalizing flow models are highly architecturally constrained (bijective mapping) and do not permit representational flexibility of high-dimensional images. PatchCore was proposed by Roth et al. (27) and builds a coreset memory bank on patch-level features of a pre-trained ResNet-50 backbone and uses nearest-neighbor anomaly scoring. PatchCore was able to achieve AUROC of 0.99 on MVTec-AD, the leading industrial anomaly detector, but on medical imaging, where there is fewer training data and domain shift is strong, the performance is significantly lower. In the present work we use a simplified PatchCore-style feature baseline (Section 3.12.2) as our unsupervised reference under the same train/test split. Because our implementation replaces the original patch-level memory bank with globally average-pooled layer-2 and layer-3 features, it is weaker than the published PatchCore method, and we therefore refer to it as a simplified PatchCore-style baseline throughout this manuscript. It should not be read as a reproduction of the original PatchCore result, and the margin reported in Section 4.4 should be interpreted as a margin over this simplified variant rather than over PatchCore as published.

### 2.5 Denoising diffusion probabilistic models

The DDPM framework (16) was a principled variational framework of iterative noise-based generative modelling, and it showed high-fidelity image generation using *T* = 1,000 denoising steps with a noise-predicting U-Net. Critical improvements were proposed in (17) such as: (i) a cosine noise schedule that minimizes noise variance at low timesteps, and preserves structural information; (ii) learnable variance prediction that enables adaptive step sizes; and (iii) a hybrid loss objective that combines simplified MSE with the variational lower bound, which significantly improves log-likelihood and samples. The diffusion sampling was redefined (19) as an implicit probabilistic model (DDIM) with an ODE-based deterministic sampler, allowing not only to sample quickly (50 steps instead of 1,000) but, more importantly, invertible deterministic encoding, which is the mathematical basis of our anomaly detector. It was shown that by including classifier gradients at every denoising step, it is possible to have fine-grained conditional control over the generated distribution to produce class-directed image synthesis, which outperforms GANs on FID and precision-recall metrics (28).

### 2.6 Classifier-free guidance

Ho and Salimans (29) proposed classifier-free guidance (CFG) as an alternative to classifier-guided diffusion that integrates conditioning directly into the diffusion model architecture by randomly dropping class labels during training (with probability p_uncond = 0.1–0.2) and linearly combining conditional and unconditional score estimates at inference. CFG eliminates the need for a separately trained noise-conditioned classifier, reduces the risk of adversarial gradient artefacts, and simplifies deployment. In MammoDiff *V*_2_, we adopt CFG jointly with explicit classifier guidance, using CFG for DDPM training and explicit classifier gradients during guided denoising inference, providing complementary conditioning signals at different stages of the pipeline. We use both signals because they operate at different stages of the pipeline: classifier-free guidance shapes the conditional score during DDPM training, ensuring the conditional and unconditional score estimates are jointly well-calibrated, while explicit classifier guidance at inference provides the spatial pixel-level gradient signal required to steer the denoising trajectory toward the healthy class manifold.

In MammoDiff V2, we propose a hybrid conditioning framework that synergistically combines Classifier-Free Guidance (CFG) and Explicit Classifier Guidance. In the training stage of Denoising Diffusion Probabilistic Model (DDPM), we follow the CFG method in which class labels are randomly replaced with a fixed probability. This enables the model to learn the conditional and unconditional distributions in the same architecture, resulting in a strong internal representation of breast anatomy without the need for an external classifier at this stage. In the guided denoising inference, however, we combine the CFG-based score estimates with explicit gradients of a pre-trained, noise-robust classifier. The dual-signal approach is driven by the unique needs of medical synthesis, where a global signal (CFG) helps hold the structure together and avoids the introduction of unwanted side effects that can arise from gradient-based approaches, while a local signal (explicit classifier gradients) gives high-frequency, class-specific guidance. MammoDiff V2 combines these complementary signals to fine-tune the creation of more subtle pathological patterns like microcalcifications or architectural distortions that may not be captured by CFG alone. The direction of guidance score is calculated as in (Equation 1):

$${\hat{\varepsilon }}_{\theta }(x_{t}c)=(1+w)\varepsilon_{\theta }(x_{t},c)-w\varepsilon_{\theta }(x_{t},\theta )+s\cdot \nabla_{xt})\log\;p_{\phi }(c|x_{t})$$ (1)

where *w* represents the CFG scale and *s* denotes the explicit guidance strength, allowing for a tunable balance between image fidelity and diagnostic alignment

### 2.7 Diffusion models for medical image analysis

Rombach et al. (30) proposed Latent Diffusion Models (LDMs) which leverage diffusion in a compressed VAE latent space, allowing for high-resolution medical image synthesis at practical computational budgets, while reducing computational cost by 4×–8× while maintaining sample quality.) Pinaya et al. (33) used transformer-based LDMs to 100,000+ brain MRI volumes in the UK Biobank with AUROC of 0.89 for unsupervised detection of brain anomalies at unprecedented scale. For unsupervised anomaly detection in brain MRI, Wyatt et al. (31) introduced AnoDDPM which used multi-scale simplex noise instead of Gaussian noise in the standard DDPM and achieved AUROC of 0.79 without a classifier. Bercea et al. (32) presented FLAIR (Feature-Level Anomaly Inversion via Reconstruction), a method for anomaly detection in generalized pathology types in multiple anatomical domains.

### 2.8 The foundational framework

One of the earliest diffusion-based medical anomaly detection frameworks is the work of Wolleb et al. (18), which is the direct predecessor of MammoDiff. They train a class-conditional DDPM and a noise-conditioned binary classifier on unpaired collections of healthy and diseased images, with only image-level labels. The DDIM reversed-ODE encodes the anatomical information in the input image into a noisy image $x_{L}$, and the DDIM denoising process is guided to the healthy class h by classifier gradient guidance, resulting in a counterfactual healthy image $x_{0}^{\prime }$. The anomaly map is the difference $\left|x_{0}-{x^{\prime }}_{0}\right|$. Their approach yielded an AUROC of 0.98 and 0.87, and Dice scores of 0.62 and 0.86 on BRATS (brain MRI, *T* = 1,000, 2D axial slices) and CheXpert (chest x-ray), respectively, demonstrating good domain-agnostic performance. In this work, MammoDiff extends this framework to mammography, proposing V2 (cosine schedule + CFG), the HSI metric, and systematically validating it on a clinically acquired Middle Eastern dataset for the first time, as far as we know (18).

Tables 1a, 1b show that there is a large divide in the existing literature. On the other hand, although general medical anomaly detection (AD) frameworks (Table 1a) have progressed from simple autoencoders to more complex diffusion-based models, they have been validated in limited datasets, such as brain MRI and chest x-ray (e.g., BraTS, CheXpert). Some of these techniques have poor reconstruction quality or fail to effectively incorporate class-conditioning, and importantly, have not been thoroughly optimized for the highly textured images of mammography. In contrast, well-known mammography-specific models (Table 1b) continue to rely on large fully-supervised training sets that frequently include more than 70,000 or 1 million expertly annotated images to attain radiologist-level accuracy. This dependency results in a “supervision bottleneck” and makes such systems hard to implement in resource-limited clinical environments or to be adapted to different populations where vast amounts of annotated data are not available. Moreover, as demonstrated in recent external validations, there is a significant performance gap when supervised models are used in domains different from the ones they were trained on. In this regard, a need for hybrid frameworks is arising that can incorporate the unsupervised generative capability of diffusion models, specifically for mammography, avoiding the need for comprehensive pixel-level annotations while ensuring a high diagnostic performance in challenging clinical environments.

### 2.9 Literature search strategy and scope of the novelty claim

Because this work states a priority claim, we describe here the search on which that claim rests. We searched PubMed, IEEE Xplore, Scopus and arXiv for records published between January 2020 and May 2026, using combinations of the terms (“diffusion model” OR “denoising diffusion” OR DDPM OR DDIM) AND (mammograph* OR “breast imaging”) AND (“anomaly detection” OR “weakly supervised” OR unsupervised). Titles and abstracts were screened by the first author, and the reference lists of the retrieved diffusion-based anomaly detection papers were checked manually for further records. The search returned only a small number of studies applying diffusion models to mammography, and the ones we retrieved use them for image synthesis, data augmentation, or fully supervised classification, rather than for classifier-guided counterfactual anomaly detection under image-level supervision only.

We therefore state the novelty claim in a deliberately bounded form: to the best of our knowledge, and within the scope of this search, no previous study has applied classifier-guided DDIM reconstruction to weakly supervised mammographic anomaly detection on a clinically acquired Middle Eastern cohort. We do not claim novelty for the underlying machinery. DDIM inversion (19), classifier guidance (28) and counterfactual healthy reconstruction for medical anomaly detection (18) are all established, and MammoDiff is in essence a domain transfer of that established pipeline. The elements we regard as genuinely new are the domain-specific characterisation of the noise level and gradient scale for mammographic tissue, and the Healthy-Shift Index. A reader may reasonably regard the first of these as an application contribution rather than a methodological one, and we would not dispute that reading.

### 2.10 Broader context in AI-assisted medical diagnosis

The methods discussed above sit within a much wider body of work on deep learning for computer-aided diagnosis, and it is useful to situate MammoDiff within that broader landscape. In breast imaging outside mammography, Butt et al. (40) reported an enhanced deep neural network ensemble for breast cancer detection in ultrasound that outperformed both a Vision Transformer and conventional transfer learning, illustrating how ensembling can partly compensate for limited data in a modality with high operator variability. Comparable ensemble and feature-selection strategies have been applied across other diagnostic domains: skin lesion classification has been studied both as a comparison of classical machine learning against deep architectures (41) and as an efficient deep learning pipeline (42); Butt et al. (43) proposed a multi-scale deep learning approach for the early detection of Alzheimer's disease from fMRI; Rehman et al. (44) applied advanced machine learning classifiers to coronary heart disease risk assessment; Khan et al. (45) addressed burn depth classification and segmentation using a deep convolutional network; and Ur Rehman et al. (46) introduced a hybrid ensemble with dynamic feature selection for thyroid disease diagnosis.

Two observations from this literature are relevant to the present study. First, almost all of these systems are fully supervised and are trained on datasets with reliable per-case labels, which is precisely the constraint that motivates the weakly supervised formulation adopted here. Second, most of them report discrimination performance well above what MammoDiff achieves. That gap is expected rather than surprising, because those tasks are supervised classification problems whereas anomaly detection with image-level labels alone is a substantially harder setting, and we do not present our AUROC as competitive with them.

## 3 Methodology

The entire framework of MammoDiff is presented in five parts: the KHCC-Mammo dataset and preprocessing pipeline (Section 3.1); the denoising diffusion probabilistic model backbone and noise schedule variants (Sections 3.2–3.3); the U-Net architecture and noise conditioned classifier (Sections 3.4 and 3.5); the inference pipeline consisting of deterministic DDIM encoding, denoising by a classifier, and computation of an anomaly map (Sections 3.6–3.8); and the evaluation protocol with the proposed Healthy-Shift Index and baseline comparisons (Sections 3.9–3.11).

### 3.1 Dataset, labels, and patient-level split

The KHCC-Mammo dataset consists of a collection of full-field digital mammography (FFDM) images from the King Hussein Cancer Center (KHCC) in Amman, Jordan, the largest oncological center in the Middle East, from 2014 to 2025. The images were obtained from GE Healthcare FFDM systems. The study received approval from the KHCC Institutional Review Board (IRB approval number: 25KHCC241F); patient consent was waived in this study, which was conducted under the retrospective de-identification protocol.

The full clinical archive available at KHCC comprises 3,013 images from 806 unique patients were selected by applying the inclusion and exclusion criteria described below. The selection of images and labelling were done before the training and evaluation of models. Figure 1 shows the characteristics of the data sets: the distribution of BI-RADS labels, the breast density profile, the composition of examination types, the time range of the data sets (2014–2025), and the prevalence of calcifications by category in the KHCC-Mammo cohort.

- ***Label adjudication:*** All mammograms were interpreted and labeled by experienced radiologists at KHCC using the ACR BI-RADS® 5th Edition lexicon (37). For every examination the report records a per-breast characterisation of the findings as benign or malignant, alongside the BI-RADS assessment category. The binary class label used for training and evaluation in this work is that reported characterisation: an examination was assigned to the Malignant class if either breast was characterised as malignant in the report, and to the Normal class otherwise. The BI-RADS assessment category is reported in Figure 1a as a characteristic of the cohort, and was not itself used to derive the class label.

**Figure 1:**
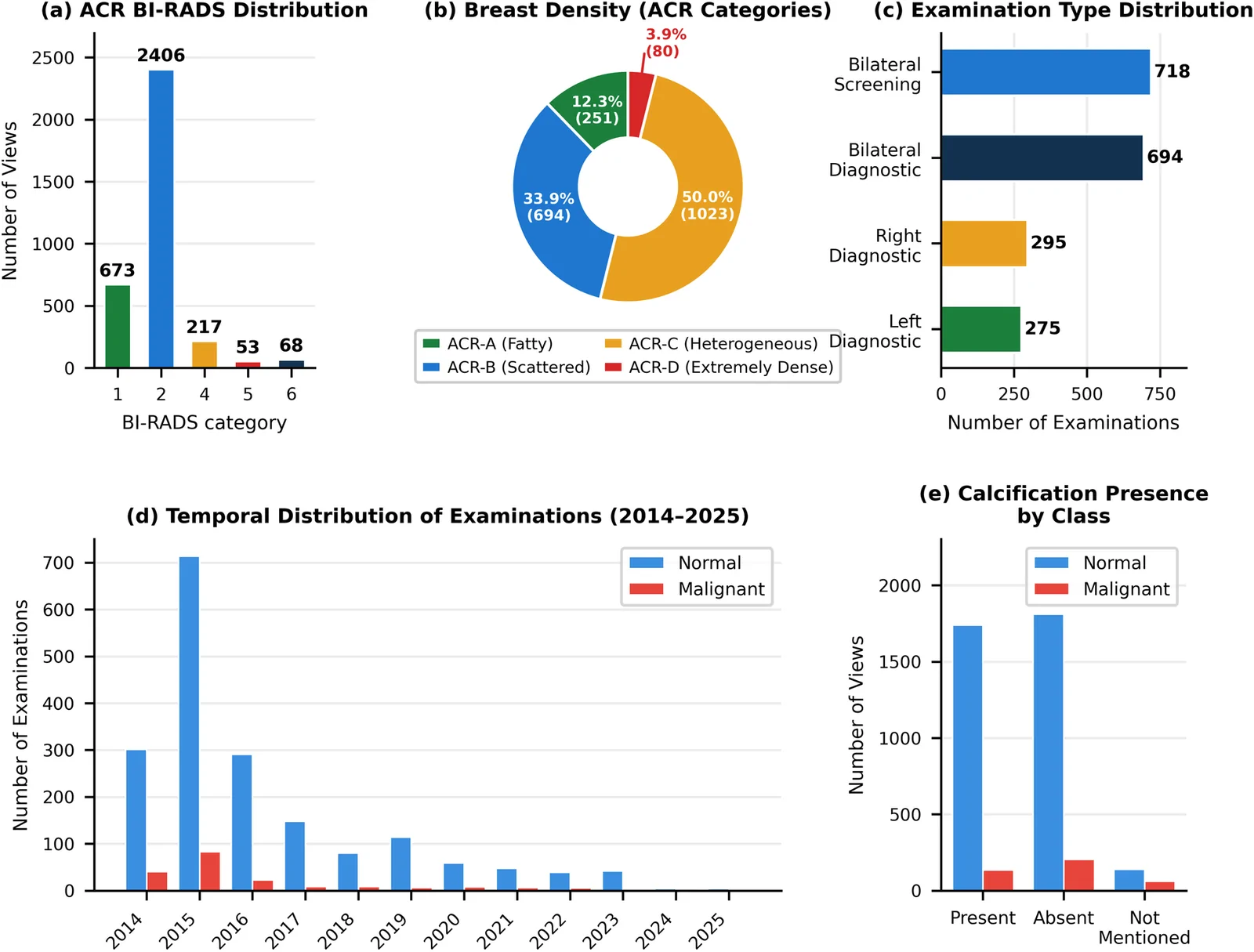
KHCC-Mammo dataset characteristics. **(a)** ACR BI-RADS distribution across all included views; categories 0 and 3 were excluded per the dataset inclusion criteria. **(b)** Breast density distribution by ACR category, reflecting the clinical density profile of the KHCC patient population. **(c)** Examination type distribution across bilateral screening, bilateral diagnostic, and unilateral diagnostic modalities. **(d)** Temporal distribution of examinations by class across the 2014–2025 acquisition period, demonstrating longitudinal clinical diversity. **(e)** Calcification presence by class across both breast sides; "Not Mentioned" reflects examinations where calcification status was not documented in the radiological report.

Two properties of this reference standard should be stated plainly, because they bound what the metrics in Section 4 can be taken to mean. First, the label is the reporting radiologist's characterisation and was not systematically linked to histopathology for this cohort. At KHCC the diagnostic pathway does resolve suspicious findings further, through biopsy where it is performed and through follow-up imaging where it is not, but that resolution was not available as a structured field for the examinations used here. The label is therefore a reported radiological characterisation and not a pathological one, and we use the word Malignant in that sense throughout. Second, the characterisation is recorded for the examination as a whole and reflects the radiologist's overall assessment of the case, which in some instances rests on documented disease history rather than on a finding visible in the examination being reported; post-treatment surveillance is the clearest example. Where that occurs, images that contain no current lesion inherit a positive label. We are not able to quantify the size of this effect for the study cohort, because the resolution of each characterisation into current finding as against documented history is not a structured field. Both properties place a ceiling on any discriminative metric computed on this cohort that is independent of the model, and both are addressable by re-deriving the labels from linked pathology outcomes and from the finding recorded for the current examination; this is listed in Section 5 as the change that would most improve the reference standard.

- ***Inclusion criteria:*** FFDM exams, female patients, bilateral or unilateral screening and/or diagnostic mammograms, CC and/or MLO projections, BI-RADS 1, 2, 4, 5, 6 at examination level.
- ***Exclusion criteria.*** Examinations that are either BI-RADS 0 or 3; images with corrupted DICOM files or missing radiological reports; post-procedural examinations that do not have interpretable mammographic findings.
- ***View composition:*** The dataset contains the examination types bilateral screening (bilateralscreening), bilateral diagnostic (bilateraldiagnostic), right diagnostic (rightdiagnostic) and left diagnostic (leftdiagnostic). The mammographic images were analyzed one by one, regardless of whether they were a CC or MLO, left or right breast, as the mammographic image was the independent sample of the image-level binary label assigned at the examination level.

This choice has a consequence that should be stated explicitly. Where disease is confined to one breast but both breasts are imaged, the views of the unaffected breast inherit the positive label of the examination even though they contain no suspicious finding. This is label noise intrinsic to weak supervision without lesion localisation, and it is the same mechanism, acting at a coarser scale, that we quantify for image patches in Section 4.9, where propagating an image-level label to four patches caps the guidance classifier well below the accuracy reached by the whole-image classifier. The reports do record a separate characterisation for each breast, so a laterality-specific labelling would remove this particular source of noise without requiring any new annotation. Adopting it is the first change we would make before a further training run, and it is listed in Section 5.

- ***Breast density distribution:*** The curated dataset is representative of the breast density distribution of the KHCC clinical population, which is heterogeneously dense (ACR category C, ∼50%), scattered fibroglandular tissue (ACR category B, ∼34%), fatty (ACR category A, ∼12%), and extremely dense (ACR category D, ∼4%), similar to the population-level density distribution reported for Middle Eastern mammographic cohorts (20).
- ***Dataset partitioning at the patient level:*** To ensure robust model evaluation and strictly prevent data leakage, the dataset was partitioned entirely at the patient level. The cohort of 806 patients was systematically allocated to the different sets, guaranteeing that no single patient's images appeared in more than one subset. Furthermore, this partitioning was stratified based on malignancy status to maintain a balanced distribution of diagnostic classes across all sets.

The resulting distribution yielded the following cohorts:

- **Training Set:** Approximately 655 patients, comprising 2,467 images (1,243 Normal, 1,224 Malignant).
- **Validation Set:** Approximately 87 patients, comprising 242 images (133 Normal, 109 Malignant).
- **Held-out Test Set:** Approximately 64 patients, comprising 304 images (151 Normal, 153 Malignant).

A further characteristic of the cohort should be stated here because it constrains several of the analyses that follow. KHCC-Mammo carries image-level binary labels derived from the BI-RADS assessment, together with examination-level metadata (view, laterality, examination type, ACR density category, and calcification mention). It does not carry pixel-level lesion masks, lesion bounding boxes, or a structured lesion-type field distinguishing mass from calcification-dominant disease. This is the reason no quantitative localisation metric is reported in Section 4.7 and no lesion-type-stratified performance breakdown is reported in Section 4.5; neither is computable from the available annotation. Obtaining a lesion-annotated subset is the single change to the dataset that would most improve the evaluation, and it is the first item of future work in Section 5.

### 3.2 Preprocessing and augmentation

A multi-stage preprocessing and data augmentation pipeline was used to ensure the robustness of the proposed model and to standardize the heterogeneous nature of the mammographic images obtained from the King Hussein Cancer Center (KHCC).

#### 3.2.1 Image preprocessing

The raw full-field digital mammography (FFDM) images may also have differences in resolution, orientation, and contrast. These differences were minimized by the following steps:

- **Image Resizing:** All images were resized to 256 × 256 pixels following the standard DDPM training protocol of (16, 18). We acknowledge that this resolution substantially attenuates high-frequency microcalcification signal mammographic microcalcifications typically measure 0.1–0.5 mm, spanning only 1–3 pixels at 256 × 256, and represents a fundamental limitation of the current implementation. This resolution choice is expected to contribute to the 40% false-negative rate observed at the Youden operating point, particularly for DCIS and clustered microcalcification presentations. A higher-resolution evaluation at an effective 512 × 512 using patch-based processing is reported in Section 4.9.

It is worth making the scale argument explicit, because it bounds what any method operating at this resolution can be expected to detect. A full-field digital mammogram from the systems used here is acquired at roughly 2,500–3,300 pixels along the longer axis, so resizing to 256 × 256 represents close to a tenfold linear reduction. An individual microcalcification of 0.2 mm, which is a clinically relevant size, occupies on the order of two to three pixels in the native image and therefore falls below a single pixel after downsampling; a cluster of such calcifications is reduced to a small local change in mean intensity rather than a resolvable pattern. Masses and architectural distortions, which are low-frequency and span tens of millimetres, survive the reduction far better. We would therefore expect the performance reported in Section 4 to be driven mainly by mass-like findings, and we would not expect the present configuration to detect calcification-only disease reliably. This is a limitation of the input representation rather than of the diffusion framework itself.

- **Pixel Normalization:** Pixel intensity values were normalized to the range [–1, +1 ] by dividing each pixel value by 255. This process accelerates gradient descent convergence during the training phase by ensuring that the input features are on a similar scale. Pixel intensity values are normalized to the range [–1, +1] as defined in (Equation 2):
  $$x^{\prime }=\left(\frac{x}{255.0}\right)\times 2.0-1.0$$ (2)

#### 3.2.2 Data augmentation

Given the complexity of mammographic patterns and to improve the model's generalization capabilities, data augmentation was employed. This technique artificially expands the training dataset, helping the network become invariant to irrelevant spatial transformations and reducing the risk of overfitting. The following transformations were applied:

- **Geometric Transformations:** To simulate different clinical positioning and anatomical variations, images were subjected to horizontal and vertical flipping, along with a random rotation of up to 20.
- **Spatial Scaling: A zoom range** of 0.2 was applied, allowing the model to recognize malignant features at varying scales and distances within the breast tissue.

### 3.3 Mammodiff overview

At test time, MammoDiff is used as a four-phase inference pipeline for each input mammogram *x*_0_. In Phase I, the input mammogram is deterministically encoded into a noisy latent representation $x_{L}$ through the reversed DDIM ordinary differential equation (Equation 14), which is run *L* times. *L* regulates the depth of encoding: higher *L* values will result in more loss of the original image structure, which will allow the decoding process to be guided more freely to alter the pathology-related areas, but with increased decoding time. The noisy latent $x_{L}$ is then decoded back to image space using *L* guided denoising steps in Phase II. In every step *t* the DDPM noise estimate *ε_θ_* is updated by the gradient of the noise-conditioned classifier *C_φ_* with respect to the current noisy image, multiplied by the classifier gradient scale *s* (Equation 15). This gradient signal moves the denoising trajectory away from the malignant feature configurations and towards the healthy class manifold, obtaining a pseudo-healthy reconstruction $x_{0}^{\prime }$. In Phase III, the anomaly map $a(i,j)=|x_{0}(i,j)-{x^{\prime }}_{0}(i,j)|$ is calculated as the absolute difference between the input image and the reconstruction, followed by spatial mean pooling over the entire mammographic image to get the image-level anomaly score *A*(*x*_0_) (Equations 17–18). Regions where the classifier guidance induced large reconstruction modifications produce high anomaly intensity, providing qualitative spatial visualisation of the detection signal. In Phase IV, the Healthy-Shift Index HSI(*x*_0_) = *C_φ_*(malignant|*x*_0_, *t* = 0) − *C_φ_*(malignant|*x*_0_′, *t* = 0) is computed by evaluating the noise-conditioned classifier at *t* = 0 on both the original and reconstructed images, yielding an internal consistency metric that is orthogonal to the pixel-level anomaly score. The two free parameters of the pipeline, noise level *L* and classifier gradient scale *s* are selected via a 3 × 3 validation-set ablation grid as described in 3.9. Figure 2 provides a schematic overview of the complete pipeline.

**Figure 2:**
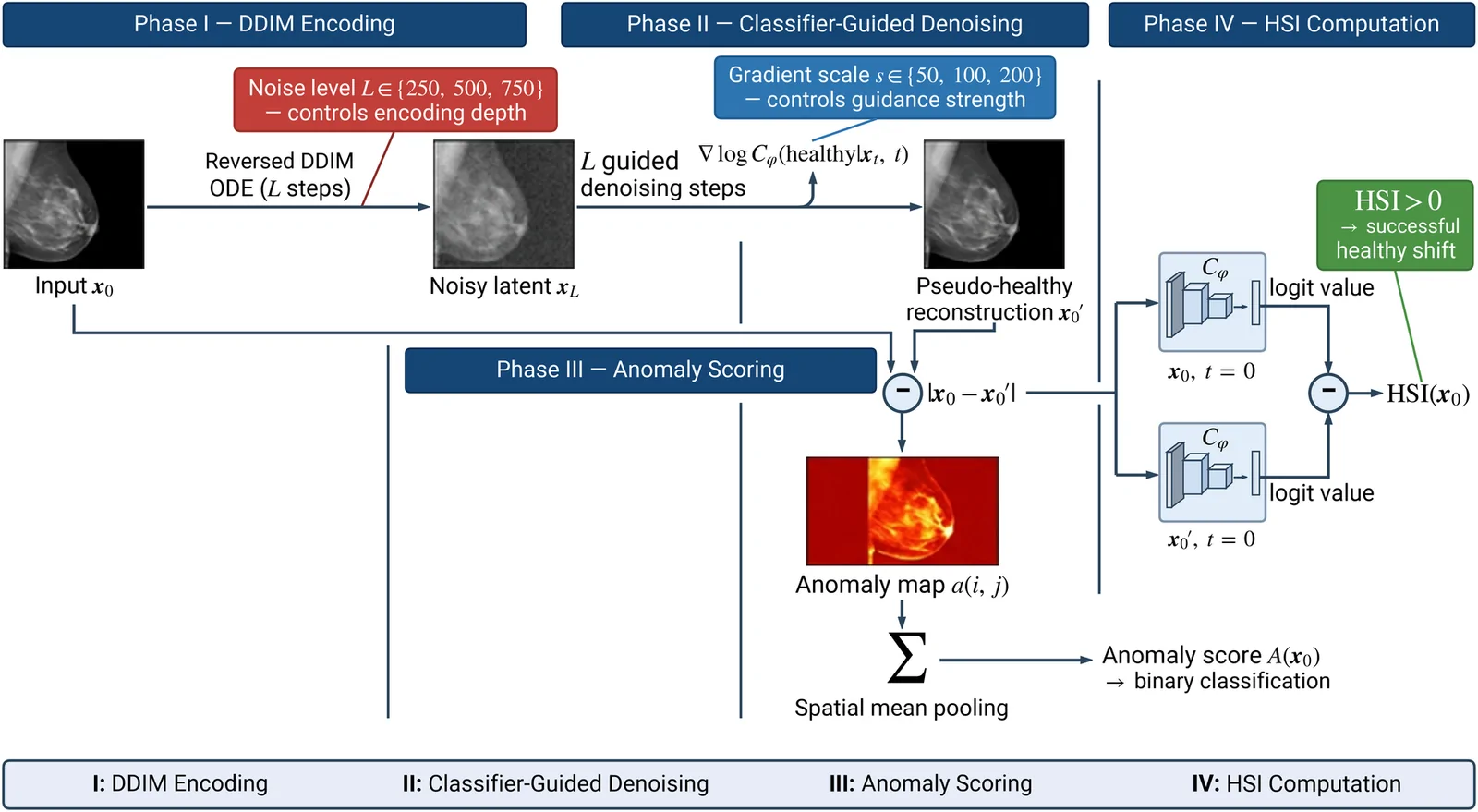
Schematic overview of the MammoDiff pipeline.

### 3.4 Diffusion backbone and conditioning

The generative backbone of MammoDiff follows the DDPM formulation of (16), extended with the architectural refinements of (17). We chose a DDPM backbone over GANs and VAEs for three principled reasons. First, training stability: DDPM optimizes a simple noise-prediction objective via mean-squared error, entirely avoiding the adversarial minimax dynamics that cause mode collapse and discriminator saturation in GAN-based mammographic synthesis. Second, reconstruction fidelity: the iterative, score-based denoising process produces fine-grained reconstructions whose qualitative tissue texture is in our visualized examples, consistent with the surrounding healthy parenchyma, a property we hypothesize contributes to the spatial coherence of the anomaly maps observed in 4.6. Third, principled noise encoding: the analytically tractable forward process (Equation 3) and the invertible DDIM ordinary differential equation (ODE) formulation (Equation 14) together enables deterministic encoding and approximate reconstruction of the input image's anatomical information into a noisy latent representation, the foundational mechanism of the anomaly detection pipeline.

The forward diffusion process q corrupts *x*_0_ over *T* = 1,000 timesteps by adding scaled Gaussian noise at each step, given by (Equation 3)

$$q(x_{t}|x_{t-1}):=N(x_{t};\surd (1-\beta_{t})x_{t-1},\beta_{t}I)$$ (3)

where $\left\{\beta_{1},\ldots ,\beta_{T}\}\in (0,1\right)$ is the noise schedule. Exploiting the Gaussian reparameterization identity, the marginal distribution at arbitrary timestep t is expressed in closed form in (Equation 4), bypassing sequential simulation of the Markov chain (16):

$$x=\surd {\bar{\alpha }}_{t}\cdot x_{0}+\surd (1-{\bar{\alpha }}_{t})\cdot \varepsilon ,\varepsilon ∼N(0,I)$$ (4)

where $\alpha_{t}:=1-\beta_{t}\;\mathrm{and}\;{\bar{\alpha }}_{t}:=\prod_{s=1^{t}}\cdot \alpha_{s}$ denotes the cumulative noise product at step t. This closed-form expression serves two critical roles in the framework: it enables efficient training by sampling noisy images at arbitrary timesteps without iterative forward simulation, and it provides the q-sampling operator used to construct the training distribution for the noise-conditioned classifier described in Section 3.5.

The reverse denoising process p*_θ_* is parameterized by a U-Net *ε_θ_* trained to predict the added noise via the simplified mean-squared error objective, formulated as (Equation 5) (16):

$$L_{\mathrm{simple}}:=\|\varepsilon -\varepsilon_{\theta }(\surd {\bar{\alpha }}_{t}x_{0}+\surd (1-{\bar{\alpha }}_{t})\varepsilon ,t)\;{\|}_{2}^{2},\varepsilon ∼N(0,I)$$ (5)

The hybrid loss of (17) augments $L_{simple}\;$ with a variational lower bound term $L_{vlb}$ as shown in (Equation 6):

$$L_{\mathrm{hybrid}}=L_{\mathrm{simple}}+\lambda \cdot L_{vlb},\lambda =0.001$$ (6)

Variance terms $\sigma_{\theta }^{2}$ are fixed to *β_t_* following the original DDPM formulation; learned variance was not implemented in the present work.

### 3.5 Noise-conditioned classifier

The noise schedule {*β_t_*} determines the rate at which structural information is corrupted across the *T* diffusion timesteps and has direct implications for both training dynamics and inference behavior. We investigate two noise schedule designs. V1 employs the canonical linear schedule; V2 employs the cosine schedule with classifier-free guidance, evaluated as a schedule ablation. Full V2 results are reported in the Supplementary Material.

#### 3.5.1 V_1_: linear schedule

The linear schedule of (16) defines the per-step noise variances as a uniform interpolation between two boundary values scaled to the number of timesteps defined as (Equation 7):

$$\beta_{t}^{\mathrm{linear}}=\left(\frac{1,000}{T}\right)\times \mathrm{linespace}\;(0.0001,0.02,T)$$ (7)

This schedule adds noise at a constant rate across all timesteps, reaching near-complete signal corruption (*ᾱ_t_* ≈ 0) at *t* = *T*. The linear schedule is the canonical configuration evaluated by (18) on BRATS2020 and CheXpert and constitutes the V1 baseline of MammoDiff.

#### 3.5.2 V_2_: cosine schedule

As a schedule ablation, we also implemented V2 incorporating the cosine noise schedule (17) and classifier-free guidance. In our experiments, V1 with the linear schedule outperformed V2 on the KHCC-Mammo test set. Full V2 implementation details, ablation results, and diagnostic analysis are deferred to the Supplementary Material. The cosine schedule defines the cumulative noise product *ᾱ_t_* and its corresponding per-step variance *β_t_* via clipping of the first-order difference, as given in (Equations 8 and 9):

$$\alpha_{t}^{-\cos}=\frac{\;f(t)}{\;f(0)}\cdot f(t)=\cos^{2}\;\left(\frac{\pi }{2}\cdot \frac{t/T+S}{1+s}\right)$$ (8)

$$\beta_{t}^{\cos}=\mathrm{clip}\left(1-\frac{{\bar{\alpha }}_{t}}{{\bar{\alpha }}_{t-1}},0,0.999\right)$$ (9)

where *s* = 0.008 is a small offset preventing *β_t_* from becoming negligibly small near *t* = 0 and thereby destabilizing the diffusion process. The cosine schedule has √*ᾱ_t_* ≈ 1.0 for *t* ≪ *T*, so the forward process introduces minimal noise in the initial timesteps, and the tissue density gradients and tissue structural boundaries which contain the best diagnostic information in mammographic images are retained. In our experiments, however, the cosine schedule did not outperform the linear schedule on the KHCC-Mammo task; full comparative results are reported in the Supplementary Material. This property is especially relevant to mammography, since the principal radiographic evidence of ductal carcinoma *in situ* is microcalcification, which appear exactly as high-frequency texture variations, which are disproportionately corrupted by linear-schedule noise at low *t*. Motivated by the potential preservation of low-noise structure, we evaluated the cosine schedule as an alternative to the linear schedule. Our empirical results indicated that the linear schedule yielded higher AUROC on the KHCC-Mammo cohort (Supplementary Material).

### 3.6 DDIM encoding and classifier-guided healthy reconstruction

The DDPM backbone employs the class-conditional U-Net architecture of (28), adapted for single-channel grayscale 256 × 256 mammographic inputs. The architecture utilizes 128 base channels with channel multipliers (1, 1, 2, 2, 4, 4) at successive down sampling scales, self-attention at 16 × 16 spatial resolutions with a single attention head, and residual blocks with GroupNorm-32 normalization and SiLU activations, yielding a total of 113,670,786 trainable parameters. Temporal position is encoded using sinusoidal timestep embeddings (38), as in (Equation 10):

$$\gamma (t)=\left[\sin\;\left(\frac{t}{10,000^{\frac{2i}{d}}}\right),\cos\;\left(\frac{t}{10,000^{\frac{2i}{d}}}\right)\right]$$ (10)

Class conditioning is implemented by adding learnable label embeddings to the timestep embedding, following the formulation of (28) in (Equation 11):

emb(t,c)=MLP(γ(t))+Wc⋅c,c∈{0,1} (11)

where *Wc*∈ℝ^{*d* × 2}^ is a learned embedding matrix. This conditioning encourages the U-Net to model class-dependent denoising distributions, rather than modelling the mixture *p*(*x*) = *Σc p*(*x*|*c*)·*p*(*c*). During guided denoising, the model decodes deterministically toward the healthy class manifold (*c* = 0), a capability that would be undefined for an unconditional model. For the V2 variant, classifier-free guidance (29) was additionally applied during training. Full V2 implementation details and the CFG formulation are reported in the Supplementary Material.

Classifier-free guidance was hypothesised to provide a conditioning signal complementary to the explicit classifier gradient guidance at inference; however, a dedicated ablation comparing explicit classifier guidance vs. CFG vs. both was not performed in the current study and remains identified future work.

### 3.7 Noise-conditioned classifier

Following (28) and (18), we train a dedicated noise-conditioned binary classifier $C_{\phi }:(x_{t},t)\to [0,1{]}^{2}$ that operates on noisy images across all noise levels encountered during the inference trajectory. This classifier provides the gradient signal that steers the denoising process toward the healthy class manifold. The architecture is an EncoderUNet with an attention pooling head, employing 32 base channels, channel multipliers (1,1,2,2,4,4), self-attention at downscale factors {8, 16, 32}, scale-shift normalization (17), and ResBlock-based down sampling, yielding 5,452,098 trainable parameters. The noise-conditioned classifier $C_{\phi }$ produces class-conditional log-probabilities defined in (Equation 12):

$$\log\;C_{\phi }(hx_{t},t)=\mathrm{logsoftmax}(\;f_{\phi }(x_{t},t))[h]$$ (12)

where $f_{\phi }$ denotes the raw logit output of the EncoderUNet. The training objective minimizes standard cross-entropy over the joint distribution of timestep and mammographic label as follows in (Equation 13):

$$L_{\mathrm{cls}}=-E_{t∼U,(X_{0},y)∼D}[\log\;C_{\phi }(yx_{t},t)]$$ (13)

A critical design feature of the classifier training procedure is the mixed timestep sampling strategy: with probability $P_{\mathrm{low}}=0.5$, training batches sample $t\in [0,T_{\mathrm{low}}]$ with $T_{\mathrm{low}}=200$, *t* = 0 (the noiseless image) is not directly sampled during classifier training. The classifier is queried at *t* = 0 only during HSI evaluation (3.10), where both input *x*_0_ and reconstruction $x_{0}^{\prime }$ are clean images. exposing the classifier to nearly clean mammographic images and enabling it to learn the class-discriminative semantic features of the healthy and malignant distributions; with complementary probability $1\;-P_{\mathrm{low}}=0.5$, timesteps are drawn from the full range *t* ∈ [0, 1,000], ensuring meaningful gradient guidance at high noise levels. This mixed-timestep sampling strategy is essential for our pipeline. Alternative approaches, such as scoring anomalies purely from likelihood differences under classifier-free guidance, do not provide the explicit pixel-space gradient signals required to steer the denoising trajectory toward the healthy class manifold. These pixel-space gradients are the mechanism through which the denoising trajectory is steered away from pathological pixel configurations, and the mixed sampling strategy ensures their informativeness across the full noise range encountered at inference, with particular emphasis on low-t timesteps where the gradient most faithfully reflects semantic class differences. Classifier training used 60,000 iterations with the AdamW optimizer (*η* = 3 × 10^−4^, weight decay *λ* = 10^−4^, The final model checkpoint was selected as the iteration achieving the highest validation-set classification accuracy across the full noise-level distribution, evaluated every 5,000 iterations. Cosine annealing to 1% of initial LR), batch size 16, gradient norm clipping to unit norm, Automatic Mixed Precision (AMP FP16), and dual NVIDIA RTX 4090 Data Parallel.

### 3.8 Deterministic DDIM encoding

The core inference mechanism enabling structure-preserving anomaly detection is the deterministic encoding of the input mammogram *x*_0_ into a noisy representation $x_{L}$ via the reversed DDIM ODE discretization of (19). This stands in contrast to the naïve approach of directly sampling from the stochastic forward process $q(x_{L}|x_{0})=N(x_{L};\surd {\bar{\alpha }}_{L}x_{0},(1-{\bar{\alpha }}_{L})I)$: stochastic sampling discards the unique anatomical fingerprint of the individual mammogram, two independent samples from $q(x_{L}|x_{0})$ would produce distinct noisy realizations, leading to reconstructions that differ not only in the pathological region but throughout the entire tissue anatomy. The reversed DDIM ODE is deterministic and exactly invertible, guaranteeing that the same *x*_0_ always maps to the same $x_{L}$ and that the subsequent deterministic decoding step approximately reconstructs *x*_0_ when no classifier guidance is applied, as DDIM inversion introduces minor reconstruction artefacts at the pixel level. Classifier gradient guidance is intended to preferentially modify pathology-associated regions during decoding; quantitative validation of this spatial selectivity is identified as future work. This deterministic structure supports anatomy preservation that fundamentally distinguishes MammoDiff, and the Wolleb et al. (18) framework from which it derives, from all prior GAN and autoencoder-based anomaly detection approaches.

The reversed DDIM encoding step at each forward iteration *t* ∈ {0, …, *L* − 1} is given by (Equation 14) (19):

$$x_{t+1}=x_{t}+\sqrt{{\bar{\alpha }}_{t+1}}\left[\left(\sqrt{\frac{1}{{\bar{\alpha }}_{t}}}-\sqrt{\frac{1}{{\bar{\alpha }}_{t+1}}}\right)x_{t}+\left(\sqrt{\frac{1}{{\bar{\alpha }}_{t+1}}}-1-\sqrt{\frac{1}{{\bar{\alpha }}_{t}}}-1\right)\cdot \varepsilon_{0}(x_{t},t)\right]$$ (14)

Applying (Equation 14) iteratively for *t* ∈ {0, …, *L* − 1} encodes *x*_0_ into the noisy latent $x_{L}$. The noise level *L* governs the trade-off between reconstruction fidelity and pathological translation capacity:

- *L* = 250: Conservative encoding, fine microcalcification texture is maximally preserved but the DDIM trajectory has limited freedom to repaint pathological regions. This conservative encoding is hypothesised to favour detection of subtle, low-grade anomalies, though we did not stratify test results by lesion grade to verify this directly.
- *L* = 500: Balanced encoding, the operating point found optimal by (18) on CheXpert and BRATS2020; constitutes our primary experimental hypothesis for the KHCC-Mammo domain.
- *L* = 750: Aggressive encoding, maximum translation freedom for removing large masses, but risks destruction of diagnostically relevant anatomical landmarks such as the pectoral muscle boundary and nipple-areola complex.

### 3.9 Classifier-guided denoising and anomaly map computation

Following DDIM encoding to $x_{L}$, the inference pipeline enters the guided denoising phase, in which the deterministic DDIM reverse process (19) is augmented at each step with the gradient of the trained classifier $C_{\phi }$ with respect to the healthy class *h* = 0. At each denoising step *t* ∈ {*L*, …, 1}, the DDPM noise prediction is modified by (Equation 15):

$$\hat{\varepsilon }=\varepsilon_{\theta }(x_{t},t)-s\cdot \sqrt{1-{\bar{\alpha }}_{t}}\cdot \nabla_{x_{t}}\;\log\;C_{\phi }(h|x_{t},t)$$ (15)

where *s* is the classifier gradient scale, the principal hyperparameter controlling pathology removal strength. The gradient $\nabla_{x_{t}}\log\;C_{\phi }(h|x_{t},t)$ is computed via backpropagation through the frozen classifier and points in the direction of increasing healthy-class log-probability in the pixel space of $x_{t}$, steering the denoising trajectory away from malignant feature configurations. The modified estimate ε^ is then substituted into the standard DDIM deterministic update rule expressed in (Equation 16) (19):

$$x_{t-1}=\sqrt{{\bar{\alpha }}_{t-1}}\cdot \frac{x_{t}-(\sqrt{1-{\bar{\alpha }}_{t}}\cdot \hat{\varepsilon }}{\sqrt{{\bar{\alpha }}_{t}}}+\sqrt{1-{\bar{\alpha }}_{t-1}}\cdot \hat{\varepsilon }$$ (16)

The gradient scale *s* modulates a domain-specific tension that is particularly acute in mammography: when *s* is too small, the classifier gradient cannot overcome the strong reconstruction prior of the DDPM, and the anomaly map is uninformative because the pathological mass remains in the reconstruction; when *s* is too large, the classifier gradient dominates the denoising dynamics and introduces hallucinated artefacts at soft tissue boundaries, Qualitative visualization of this boundary-artefact effect across *s* ∈ {50, 100, 200} on the same malignant case is identified as a follow-up addition to strengthen this observation, particularly at the pectoral muscle margin and the skin-air interface, where density gradients are empirically observed to be steepest in our visualized cases. This boundary artefact problem is more pronounced in mammography than in brain MRI, where the rigid skull provides a natural spatial constraint on gradient propagation. Our ablation over *s* ∈ {50, 100, 200} provides domain-specific calibration for the KHCC dataset.

Upon completion of the *L*-step guided denoising pass, the pixel-wise anomaly map is computed as the absolute difference between the original input mammogram and the reconstructed healthy image defined as (Equation 17):

$$a(i,j)=|x_{0}(i,j)-x_{0}^{\prime }(i,j)|$$ (17)

Mammographic regions that the classifier-guided denoising identifies as pathological are repainted toward healthy-tissue appearance, yielding large anomaly values. Conversely, healthy anatomical regions are reconstructed with high fidelity, producing near-zero anomaly values. The image-level anomaly score used for binary classification is the spatial mean of the anomaly map, Alternative pooling strategies, including breast-region-mask mean, 95th-percentile intensity, and top-*k* mean may better capture concentrated pathological signals and are identified as priority follow-up in (Equation 18):

$$A(x_{0})=\frac{1}{HW}\sum_{i,j}a(i,j)$$ (18)

where *H* = *W* = 256. A classification decision is obtained by thresholding $\left(Ax_{0}\right)$ at the Youden-optimal operating point, as described in Section 3.11.

### 3.10 Healthy-shift index: a novel interpretability metric

Standard detection metrics such as AUROC and F1 quantify image-level discrimination, while Dice quantify pixel-level qualitative anomaly map when lesion masks are available. They measure whether the spatial distribution of *a*(*i*,*j*) correctly identifies the pathological region relative to the ground truth label. However, they provide no information about whether the guided reconstruction has successfully translated the malignant case toward the healthy class manifold in the semantic probability space of the trained classifier. A method could produce a high-intensity anomaly map simply because the reconstruction introduces widespread artefacts, without any meaningful semantic shift toward the healthy distribution. To disentangle pixel-level reconstruction error from semantic class translation quality, we introduce the Healthy-Shift Index (HSI) as a complementary interpretability metric.

For a given mammogram *x*_0_ with reconstructed healthy image $x_{0}^{\prime }$, HSI is defined as the change in healthy-class probability assigned by $C_{\phi }$ before vs. after guided reconstruction as shown in (Equation 19), evaluated at noise level *t* = 0 (i.e., on the clean images):

$$\mathrm{HSI}(x_{0})=C_{\phi }(h|{x^{\prime }}_{0},t=0)-C_{\phi }(h|x_{0},t=0)$$ (19)

A positive HSI for a malignant case indicates that $x_{0}^{\prime }$ is assigned a higher healthy-class probability than the input *x*_0_ confirming that the guided reconstruction successfully translated the pathological input toward the healthy distribution. Conversely, a normal case would be expected to exhibit an HSI close to zero, reflecting that the classifier assigns approximately equal healthy-class probability to the original image and to its reconstruction, because the guided process should make only minimal modifications to already-healthy anatomy. As reported in Section 4.6, this expectation is only partially met in practice, and we discuss the consequences for the interpretation of the metric there. We report three aggregate HSI statistics computed over the malignant test subset: the mean HSI $\left(\mu_{\mathrm{HSI}}\right)$, the median HSI $\left(m_{\mathrm{HSI}}\right)$, and the proportion of malignant cases achieving HSI > 0 as expressed in (Equation 20):

$$P_{\mathrm{HSI}}>0=\frac{1}{N_{\mathrm{mal}}}\sum_{i:y_{i}=1}1[\mathrm{HSI}(x_{0}^{i})>0]$$ (20)

A value of P(HSI>0)>50% supports that the classifier-guided diffusion framework is functioning as intended for the majority of malignant test cases, constituting an internal consistency check on the translation pipeline that cannot be inferred from AUROC or Dice score alone. As set out in Section 4.6, this is a check of internal consistency and not an independent validation, because the classifier that supplies the guidance also supplies the evaluation. Because HSI is computed using the guidance classifier, it validates consistency with the learned class-direction rather than independent clinical correctness. Therefore, HSI is reported alongside image-level detection and qualitative anomaly map metrics.

### 3.11 Hyperparameter ablation

Two hyperparameters govern inference behavior in the MammoDiff pipeline: the noise level *L*, which determines how deeply the input is encoded along the diffusion trajectory, and the classifier gradient scale *s*, which controls the strength of class-directed guidance during denoising. Both parameters critically influence the fidelity-translation trade-off and are expected to have domain-specific optimal values that differ from those established for brain MRI and chest radiography by (18). We therefore conduct a systematic 3 × 3 grid search:

- Noise level: *L* ∈ {250, 500, 750}
- Classifier gradient scale: *s* ∈ {50, 100, 200}

yielding 9 experimental configurations, each evaluated on the KHCC validation set. The configuration achieving the highest validation AUROC was selected as (L*, s*) and subsequently evaluated once on the held-out test set as defined in (Equation 21):

$$\left(L,s*)=\mathrm{argma}x_{\left(L,s\right)}\mathrm{AUROC}\_\mathrm{val}(A(x_{0};L,s),y\right)$$ (21)

This protocol mirrors the sensitivity analysis of (18), who evaluated *L* ∈ {250, 500, 750} and *s* ∈ {0, …, 600} on BRATS2020 and CheXpert, enabling direct cross-domain methodological comparison. The optimal hyperparameters identified for KHCC-Mammo are subsequently used for all final comparative evaluations reported in Section 4. Although AUROC was used for primary selection, HSI and qualitative reconstruction fidelity were monitored to avoid selecting configurations that improve classification at the expense of counterfactual validity.

### 3.12 Baseline methods

MammoDiff is benchmarked against two complementary reference methods: a fully supervised classifier and an unsupervised feature-based anomaly detector.

#### 3.12.1 Supervised baseline: ResNet-50

ResNet-50 (5) with ImageNet pre-trained weights is fine-tuned end-to-end on the KHCC training set. The final fully-connected classification head is replaced with a binary output layer. Training employs the AdamW optimizer (*η* = 10^−4^, weight decay *λ* = 10^−4^) with cosine annealing over 30 epochs, batch size 16, and horizontal and vertical flipping augmentation. Greyscale mammograms are replicated across three channels to satisfy the ImageNet input specification. This baseline operates under full image-level supervision, the same labelling overhead as MammoDiff, but employs a discriminative rather than generative paradigm, providing no inherent counterfactual reconstruction, any qualitative anomaly map requires *post hoc* saliency methods. It serves as a fully supervised image-level reference baseline. Best epoch was selected based on validation AUROC, evaluated after each training epoch. The Youden-optimal classification threshold was computed on the validation set and held fixed for the final test-set evaluation.

#### 3.12.2 Unsupervised baseline: simplified PatchCore-style

We use a simplified PatchCore-style feature baseline inspired by (27). Unlike the original patch-level memory bank, our implementation extracts globally average-pooled features from the outputs of ResNet-50 layers 2 and 3, producing a single embedding vector per image. The image-level anomaly score is the minimum Euclidean distance to the nearest prototype in *M* given by (Equation 22):

$$A_{\mathrm{patchCore}}(x)=\min_{m\varepsilon M}\|\phi (x)-m{\|}_{2}$$ (22)

A 10% coreset is retained via random subsampling, subject to a minimum of 100 prototypes. This simplified baseline requires only normal-class image labels to identify the normal training subset; no malignant labels are used at any stage. It provides an annotation-efficient unsupervised reference for isolating the benefit of classifier-guided counterfactual translation over diffusion reconstruction alone. A fully unguided MammoDiff variant (classifier scale = 0) was not evaluated in the current study and is identified as follow-up work.

### 3.13 Evaluation metrics

All methods are evaluated on the held-out KHCC test set. Classification performance is reported across a comprehensive set of metrics covering discrimination, accuracy, and clinical operating characteristics.

#### 3.13.1 Area under the ROC curve (AUROC)

The AUROC is the primary discriminative metric, computed as the area under the receiver operating characteristic curve tracing the true positive rate (TPR) against the false positive rate (FPR) across all decision thresholds. AUROC is threshold-independent and provides a global summary of ranking performance, formally expressed in (Equation 23):

$$\mathrm{AUROC}=\int_{0}^{1}\mathrm{TPR}(\mathrm{FP}R^{-1}(t))dt\;$$ (23)

When the raw AUROC is below 0.5, score polarity reversal is applied consistent with the convention established by (18) and (31).

#### 3.13.2 Operating thresholds and clinical operating points

All threshold-dependent metrics are evaluated at the Youden-optimal decision threshold t*, defined as in (Equation 24):

$$t^{*}=\mathrm{argma}x_{t}(\mathrm{TPR}(t)-\mathrm{FPR}(t))$$ (24)

This threshold maximizes the Youden index *J* = Sensitivity + Specificity − 1 (39). Because mammographic screening applications prioritise high sensitivity at constrained false-positive rates, sensitivity at fixed specificity operating points is identified as a clinically relevant evaluation dimension and is reported where computationally feasible in the results.

#### 3.13.3 Classification metrics

All remaining threshold-dependent metrics are computed at t* using standard confusion-matrix definitions. Accuracy measures the overall proportion of correctly classified mammograms: (TP + TN)/(TP + TN + FP + FN). Precision is TP/(TP + FP); recall (sensitivity) is TP/(TP + FN); specificity is TN/(TN + FP). The F1-score is the harmonic mean of precision and recall: 2·(Precision·Recall)/(Precision + Recall), and is particularly informative under the near-symmetric class distribution of KHCC-Mammo. Throughout the manuscript, AUROC serves as the primary metric; threshold-dependent metrics are reported at t* for clinical interpretation.

#### 3.13.4 Healthy-shift index (HSI)

For MammoDiff variants only, three HSI aggregate statistics are additionally reported over the malignant test subset: mean HSI $\left(\mu_{HSI}\right)$, median HSI $\left(m_{HSI}\right)$, and the proportion of malignant cases achieving HSI > 0, denoted *p* (HSI > 0). A positive HSI indicates that the guided reconstruction $x_{0}^{\prime }$ is assigned higher healthy-class probability than the input *x*, confirming successful semantic translation toward the healthy distribution. These statistics provide an internal consistency check on the classifier-guided translation mechanism that is orthogonal to all image-level discrimination metrics. They do not constitute independent validation; see Section 4.6.

#### 3.13.5 Statistical analysis

Uncertainty for AUROC is quantified by non-parametric bootstrap resampling of the test set with 1,000 resamples, from which we report the 2.5th and 97.5th percentiles as a 95% confidence interval. We report intervals for AUROC only, because bootstrapping a threshold-dependent metric requires the operating threshold to be re-derived within each resample, which confounds the uncertainty of the metric with the instability of the threshold. We note that bootstrap intervals for two methods evaluated on the same images are correlated, so overlapping intervals do not by themselves imply the absence of a difference, and we have accordingly avoided drawing any conclusion of that form from interval overlap alone.

For the Healthy-Shift Index we report, in addition to the aggregate statistics defined in Section 3.10, the separation between the malignant and the normal HSI distributions. This separation is quantified in two ways. First, by the difference in means and in medians between the two classes. Second, by treating the sign of the index as a binary outcome and comparing the proportion of cases with a positive HSI, for which we report a Newcombe hybrid-score 95% confidence interval for the difference in proportions, an odds ratio as the effect size, and a two-sided Fisher exact test. Fisher's exact test is preferred here because it makes no large-sample approximation, and the proportion-based comparison is reported because it operates on the same quantity, *p*(HSI > 0), that the aggregate statistics of Section 3.10 are based on.

## 4 Results and discussion

This section reports the experimental results of MammoDiff on the KHCC-Mammo dataset. We first describe the experimental setup and the validation-set hyperparameter selection protocol (4.1–4.2), followed by the single held-out test-set evaluation (4.3), a comparison with baseline methods (4.4), clinical operating point and triage analyses (4.5), and the counterfactual validity assessment via the Healthy-Shift Index (4.6).

### 4.1 Experimental setup

All experiments were conducted on two NVIDIA GeForce RTX 4090 GPUs (25.8 GB VRAM each) under PyTorch 2.× with Automatic Mixed Precision (FP16) and TF32 matrix acceleration. The DDPM backbone (*ε_θ_*, 113,670,786 parameters) was trained for 80,000 iterations with batch size 20, learning rate 10^−4^, EMA decay 0.9999, and the hybrid loss objective of (17) combining ℒ_simple (Equation 4) with the variational lower bound ℒ_vlb (Equation 5). The final DDPM checkpoint was the EMA-averaged model at iteration 80,000. The noise-conditioned classifier (*C_φ_*, 5,452,098 parameters) was trained for 60,000 total iterations with batch size 16, learning rate 3 × 10^−4^, cosine annealing, and mixed-timestep sampling; the classifier checkpoint was selected as the iteration achieving the highest validation-set classification accuracy, evaluated every 5,000 iterations. Both models employed the V1 linear noise schedule with *T* = 1,000 timesteps. Cosine schedule variants (V2) are reported in the Supplementary Material.

The KHCC-Mammo dataset comprises 3,013 mammographic images acquired from 806 unique patients at King Hussein Cancer Center. These 806 patients are those for whom the corresponding image series could be retrieved from the institutional imaging archive and matched to a structured report; the reporting archive itself covers a larger number of examinations. Dataset partitioning was performed at the patient level, ensuring strict non-overlap between splits: each of the 806 patients was assigned exclusively to one of three sets training, validation, or held-out test, such that no patient contributed images to more than one partition. This patient-level assignment ensures that all images from a given patient, including ipsilateral CC and MLO views, remain within the same split, thereby preventing intra-patient data leakage that would otherwise arise from image-level random sampling. Class-stratified allocation was applied to maintain approximately balanced malignancy ratios across all three partitions. The resulting image-level counts are: training set (*n* = 2,467 images; 1,243 Normal, 1,224 Malignant); validation set (*n* = 242 images; 133 Normal, 109 Malignant); and held-out test set (*n* = 304 images; 151 Normal, 153 Malignant). The test set was not accessed until the final single evaluation reported in 4.3; all hyperparameter selection was performed exclusively on the validation set.

### 4.2 Hyperparameter ablation on the validation set

MammoDiff introduces two hyperparameters governing the reconstruction–translation trade-off: the noise encoding level *L* ∈ {250, 500, 750} and the classifier gradient scale *s* ∈ {50, 100, 200}, yielding a 3 × 3 ablation grid of nine configurations as shown in Table 2. Each configuration was evaluated on the KHCC-Mammo validation set (*n* = 242) using AUROC as the primary selection criterion. The configuration (L*, s*) = (750, 50) achieved the highest validation AUROC of 0.7343 and was subsequently used for the final held-out test evaluation without further adjustment.

**Table 2:** Validation-set ablation grid for mammoDiff. AUROC and F1 reported on the KHCC-Mammo validation set (*n* = 242). The selected configuration (L* = 750, s* = 50) is highlighted. Monotonically increasing AUROC with *L* at *s* = 50 confirms the benefit of deeper noise encoding for the KHCC-Mammo domain.

L	*s*	Val AUROC	Accuracy	F1	Δ AUROC vs. *L* = 250	Time (min)
250	50	0.6977	0.6570	0.5990	—	102.4
250	100	0.6989	0.6570	0.6244	+0.0012	102.2
250	200	0.7022	0.6570	0.6244	+0.0045	102.2
500	50	0.7034	0.6736	0.5683	+0.0057	204.9
500	100	0.6993	0.6818	0.5600	+0.0016	263.1
500	200	0.6965	0.6736	0.5820	−0.0012	217.9
750	50	0.7343	0.6942	0.6754	+0.0366	340.1
750	100	0.7219	0.6901	0.6193	+0.0242	349.9
750	200	0.7197	0.6942	0.6667	+0.0220	332.3

Table 2 gives rise to three observations. First, AUROC monotonically increases as the noise level *L* increases at *s* = 50, ranging from 0.6977 at *L* = 250 to 0.7343 at *L* = 750, with a difference of 3.66 AUROC percentage points. This is consistent with the meaning of deeper DDIM encoding providing more freedom for the classifier guidance to repaint the pathology associated regions. Secondly, lower gradient scale (*s* = 50) achieves better performance than higher *s* in all cases, and the difference increases as *L* increases: at *L* = 750, *s* = 200 performs 1.46 AUROC percentage points worse than *s* = 50, consistent with the boundary-artefact hypothesis discussed in 3.7. Third, as expected, the per-image inference time scales linearly with *L*; at *L* = 750, the mean time required for inference per image is ∼84 s on two RTX 4090 GPUs.

### 4.3 Final test-set evaluation

The selected configuration (L* = 750, s* = 50) was evaluated once on the held-out KHCC-Mammo test set (*n* = 304 images; 151 Normal, 153 Malignant). The Youden-optimal classification threshold was computed from the validation ROC curve and applied without modification to the test set. Table 3 reports all evaluation metrics, and Figure 3 shows the corresponding ROC curve [AUROC = 0.7246, 95% CI (0.6685, 0.7825)].

**Table 3:** Final test-set evaluation of mammoDiff (L* = 750, s* = 50) on the KHCC-mammo test set (*n* = 304). AUROC reported with 95% bootstrap confidence interval (*n* = 1,000 resamples).

Metric	Value	95% CI	TP	FP	TN	FN
AUROC	0.7246	[0.6685, 0.7825]	—	—	—	—
Accuracy	0.6711	—	92	39	112	61
F1-score	0.6479	—	92	39	112	61
Precision	0.7023	—				
Recall (Sensitivity)	0.6013	—				
Specificity	0.7417	—				

**Figure 3:**
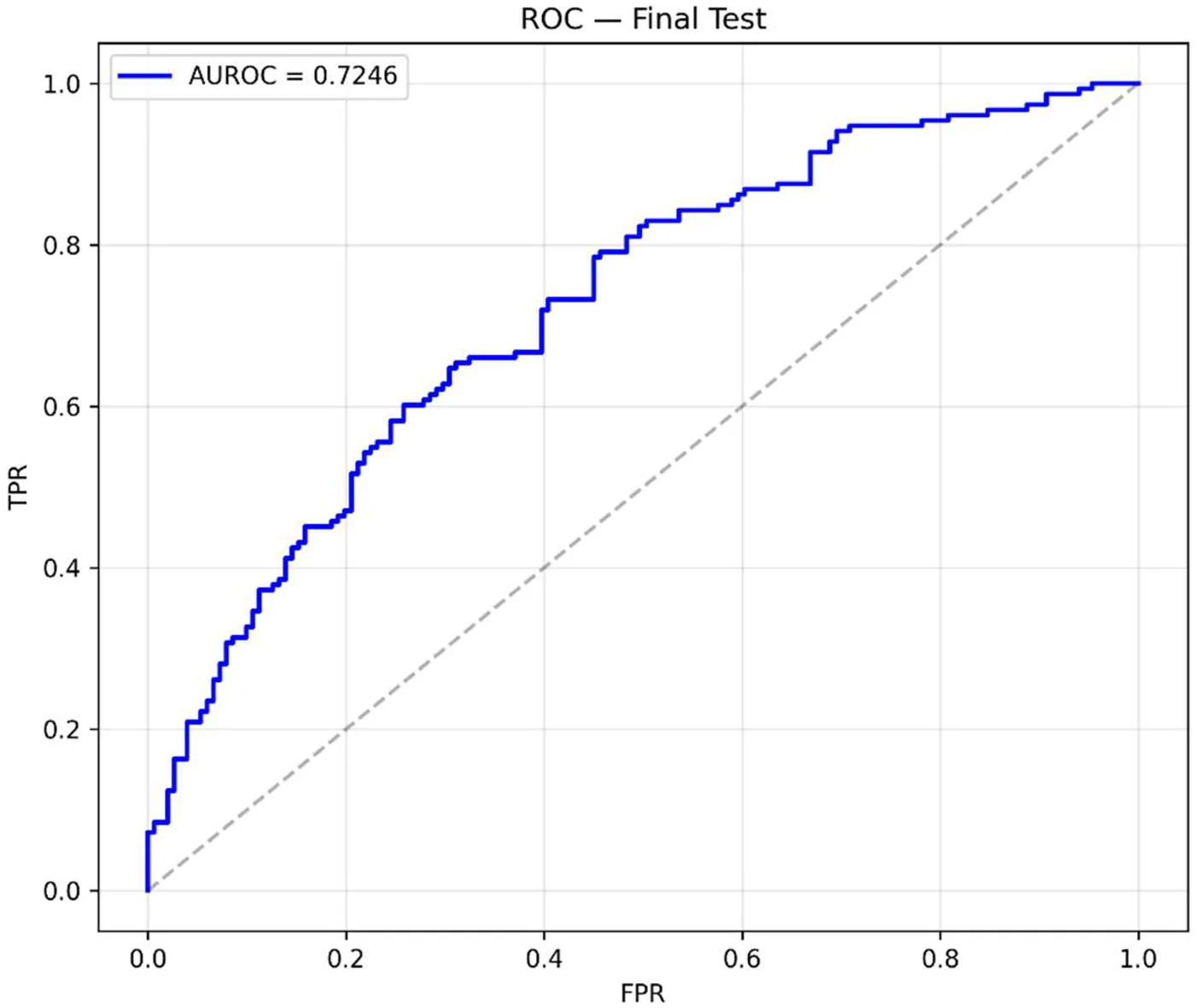
ROC curve for mammoDiff (L* = 750, s* = 50) on the KHCC-mammo held-out test set (*n* = 304). AUROC = 0.7246 [95% CI: (0.6685, 0.7825)]. The curve demonstrates meaningful discrimination above the random baseline across all operating thresholds.

MammoDiff achieves AUROC = 0.7246 on the held-out test set as a methodological proof-of-concept of weakly supervised classifier-guided diffusion applied to a Middle Eastern mammographic cohort. At the Youden-optimal operating threshold, the method attains a sensitivity of 0.6013 and specificity of 0.7417, performance that is not deployable as a clinical screening or triage tool. For context, current FDA-cleared computer-aided triage systems target sensitivity ≥87% at recall rates ≤10%, substantially exceeding the present results. The Youden-optimal threshold balances sensitivity and specificity without imposing the clinical cost asymmetry required for screening, and therefore does not represent an operationally meaningful operating point for cancer detection. The relatively higher specificity than sensitivity is characteristic of generative-reconstruction anomaly scoring, which tends to produce conservative anomaly scores. We report a bootstrap interval for AUROC only. Bootstrapping a threshold-dependent metric such as sensitivity requires the operating threshold to be re-derived within each resample, so the resulting interval reflects the instability of the threshold as much as the uncertainty of the metric itself and is not interpretable as an interval for the sensitivity reported here; we therefore do not report one. The modest test-set size (*n* = 304) nevertheless cautions against over-interpretation of any individual threshold-dependent value. The AUROC = 0.7246, as a threshold-independent summary, is the appropriate primary measure for this proof-of-concept evaluation; it demonstrates that classifier-guided diffusion reconstruction produces a meaningfully better anomaly ranking than the unsupervised baseline on this cohort, establishing a reproducible baseline for future work rather than a clinical deployment candidate.

### 4.4 Comparison with baseline methods

MammoDiff is compared against two complementary reference methods: a simplified PatchCore-style feature baseline operating in the normal-only unsupervised setting, and a supervised ResNet-50 classifier serving as a fully-labelled reference. Table 4 presents the complete multi-metric comparison; Figure 4 shows the combined ROC curves with 95% bootstrap confidence intervals for all three methods.

**Table 4:** Comparative evaluation of mammoDiff and baseline methods on the KHCC-mammo test set (*n* = 304). All AUROC values reported with 95% bootstrap confidence intervals (*n* = 1,000 resamples). Supervision level: “normal-only” indicates one-class unsupervised training; “Weakly supervised” indicates image-level binary labels only; “Supervised” indicates full image-level label supervision.

Method	Supervision level	AUROC	F1	Sensitivity	Specificity	AUROC 95% CI
Simplified PatchCore-style	Normal-only (one-class)	0.6634	0.6801	0.7712	0.4967	[0.5973, 0.7255]
MammoDiff (Ours)	Weakly supervised	0.7246	0.6479	0.6013	0.7417	[0.6685, 0.7825]
ResNet-50 (Reference)	Supervised	0.8100	0.7518	0.6928	0.8477	[0.7578, 0.8577]

**Figure 4:**
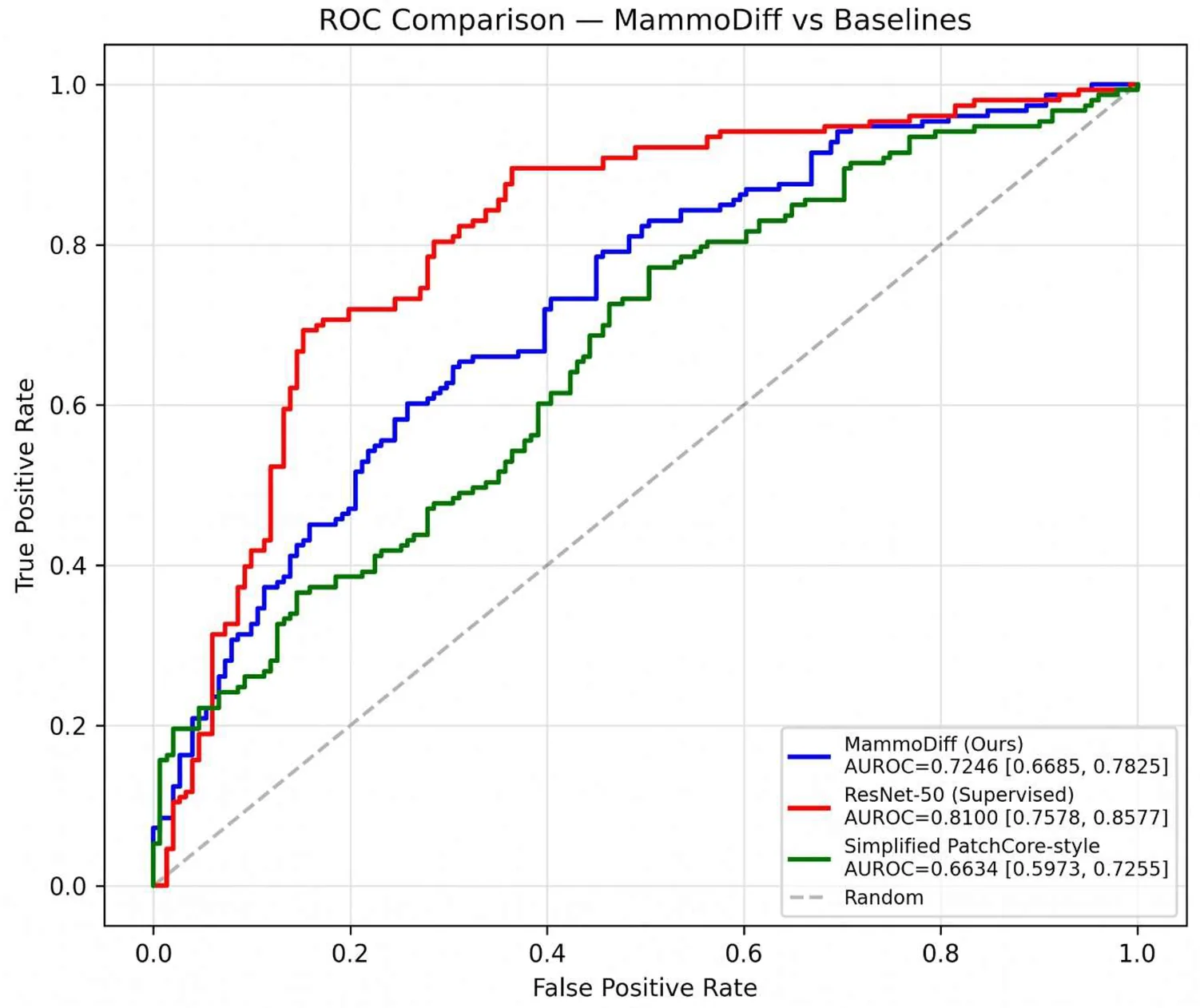
Combined ROC curves for mammoDiff, resNet-50, and the simplified patchCore-style baseline on the KHCC-mammo test set (*n* = 304). AUROC values with 95% bootstrap confidence intervals are shown in the legend. MammoDiff (AUROC = 0.7246) exceeds the simplified PatchCore-style baseline (AUROC = 0.6634) by 6.1 percentage points while requiring only image-level binary labels rather than supervised class annotations.

MammoDiff achieves AUROC = 0.7246, surpassing the simplified PatchCore-style baseline (AUROC = 0.6634) by 6.1 percentage points while operating in the weakly supervised setting without requiring class annotations during inference. The 95% confidence intervals for MammoDiff [0.6685, 0.7825] and the simplified PatchCore-style baseline [0.5973, 0.7255] overlap over much of their range. Because both methods are evaluated on the same images these intervals are correlated, so their relative position is not a test of difference, and we draw no inference about statistical significance from it. The supervised ResNet-50 reference achieves AUROC = 0.8100 [0.7578, 0.8577], reflecting the expected performance advantage of full class-label supervision, and should not be interpreted as an upper bound but as a directional reference for what full supervision provides on this dataset. The 8.5 percentage-point gap between MammoDiff and ResNet-50 is not the price of annotation-free training, since both methods use exactly the same image-level labels and neither uses lesion-level annotation. It is the price of forgoing discriminative training on those labels in exchange for a generative model that also yields a spatial visualisation. What MammoDiff avoids relative to a fully supervised detection or segmentation system is pixel-level lesion delineation, and that is the only sense in which it is more annotation-efficient. Importantly, MammoDiff provides two capabilities that ResNet-50 cannot: qualitative spatial anomaly visualisation through the pixel-wise reconstruction difference map, and semantic guidance validation through the Healthy-Shift Index (4.6), both of which are inherent to the generative counterfactual reconstruction paradigm and independent of the image-level AUROC.

The simplified PatchCore-style specificity of 0.4967 reflects a systematic threshold bias in the nearest-neighbour anomaly score distribution, which assigns elevated scores to a large fraction of normal cases. In contrast, MammoDiff achieves a specificity of 0.7417–4.5 percentage points higher indicating that the classifier-guided reconstruction produces a more calibrated anomaly score distribution that better separates normal and malignant mammograms at the global image level. This specificity advantage may be clinically meaningful in population screening contexts where false-positive recall rates directly affect patient anxiety and healthcare resource consumption.

The Average Precision results of Figure 5 are consistent with the ordering seen in AUROC, with MammoDiff above the simplified PatchCore-style baseline under the precision-recall trade-off; as above, we present this as a consistent ordering rather than as a demonstrated difference. However, it should be noted that the AP values are reported for the approximately balanced test-set class distribution (153 malignant, 151 normal) and would be significantly lower in the case of a realistic prevalence of screening in the population (typically below 1% malignancy).

**Figure 5:**
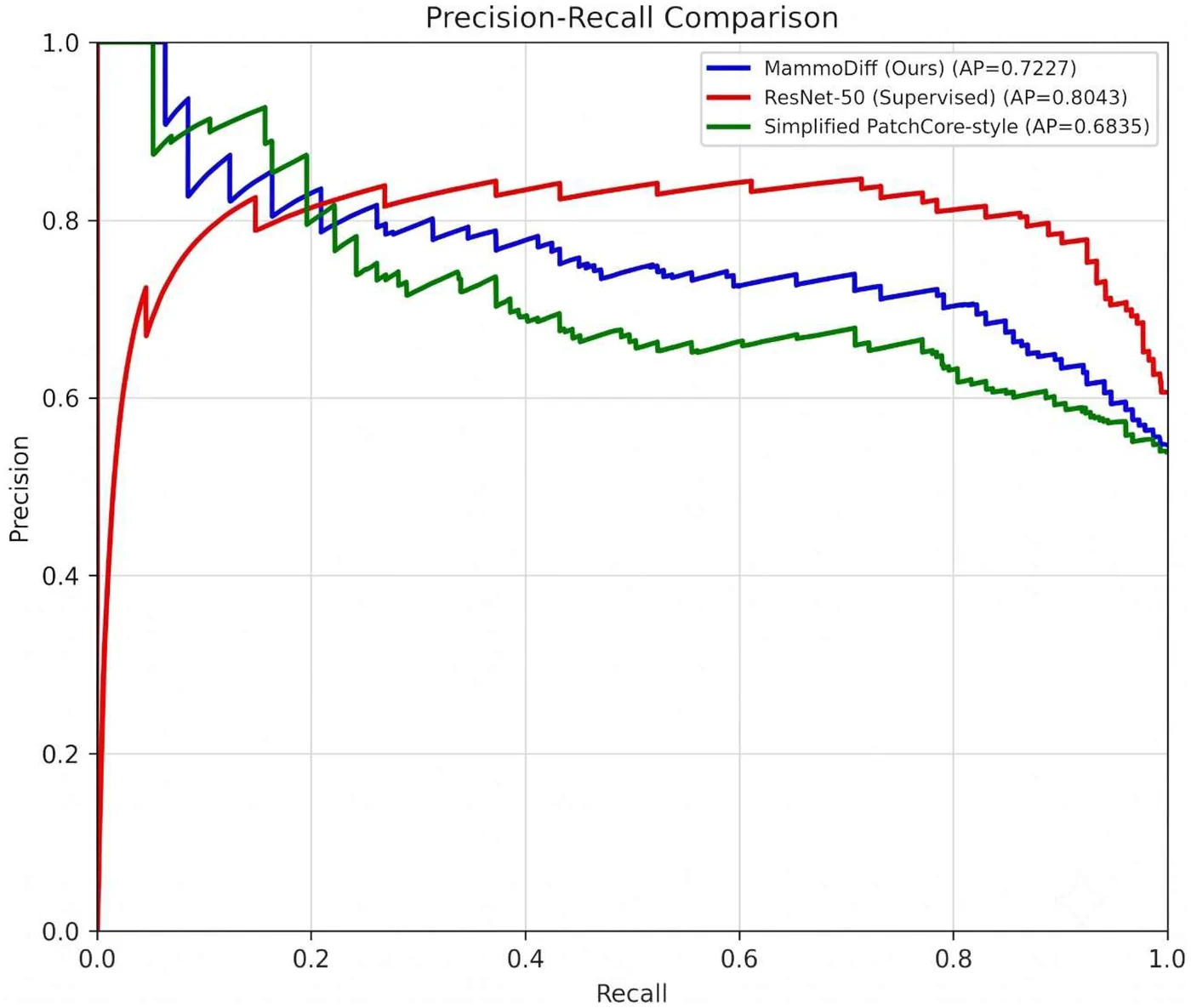
Precision-Recall curves for all three methods on the KHCC-mammo test set. Average Precision (AP): MammoDiff = 0.7227, ResNet-50 = 0.8043, simplified PatchCore-style = 0.6835. Note that all AP values are reported under the approximately balanced class distribution of the test set (153 malignant, 151 normal); precision values would differ substantially under population-level screening prevalence.

Because all three methods are evaluated on exactly the same test images, the bootstrap confidence intervals reported above are correlated, and comparing them by inspection is not a valid test of difference. We therefore do not claim statistical significance for the 6.1 percentage-point margin over the unsupervised baseline. With 304 test images and intervals of the width reported above, a margin of this size sits close to the limit of what this sample can resolve, and we present it as a consistent ordering across metrics rather than as a demonstrated difference.

It is also worth being explicit about the clinical meaning of the 8.5 percentage-point deficit relative to the supervised reference, since Reviewer comment on this point is well taken. This is not a marginal difference. In practical terms it means that at any operating point a radiologist would actually use, MammoDiff will either miss more cancers or generate more recalls than a conventional supervised classifier trained on the same images. The weakly supervised setting therefore offers no favourable trade for a centre that already holds image-level labels, because those same labels would train the supervised model. The setting in which we believe the method is defensible is different: a centre holding a large archive of images with coarse or retrospective labels, which wants spatial anomaly visualisations without paying for lesion delineation. There MammoDiff offers something the discriminative model does not, at a cost in discrimination that the present results quantify.

### 4.5 Clinical operating points and triage analysis

To assess performance at clinically constrained operating points, the primary evaluation paradigm for screening applications, Table 5 reports sensitivity at fixed specificity levels of 80%, 90%, and 95%, and specificity at fixed sensitivity levels of 80%, 90%, and 95%, extracted from the respective ROC curves without threshold re-optimization. Figure 6 presents the triage and workload curves, showing the percentage of malignant cases captured as a function of the percentage of flagged cases.

**Table 5:** Clinically constrained operating points for all three methods on the KHCC-mammo test set. Sens@Spec and Spec@Sens values extracted from the ROC curve at the target operating point.

Method	Sens @Spec 80%	Sens @Spec 90%	Sens @Spec 95%	Spec @Sens 80%	Spec @Sens 90%	Spec @Sens 95%	AP
Simplified PatchCore-style	0.386	0.261	0.222	0.437	0.291	0.099	—
MammoDiff (Ours)	0.471	0.327	0.209	0.517	0.331	0.219	—
ResNet-50 (Reference)	0.719	0.418	0.190	0.715	0.543	0.272	—

**Figure 6:**
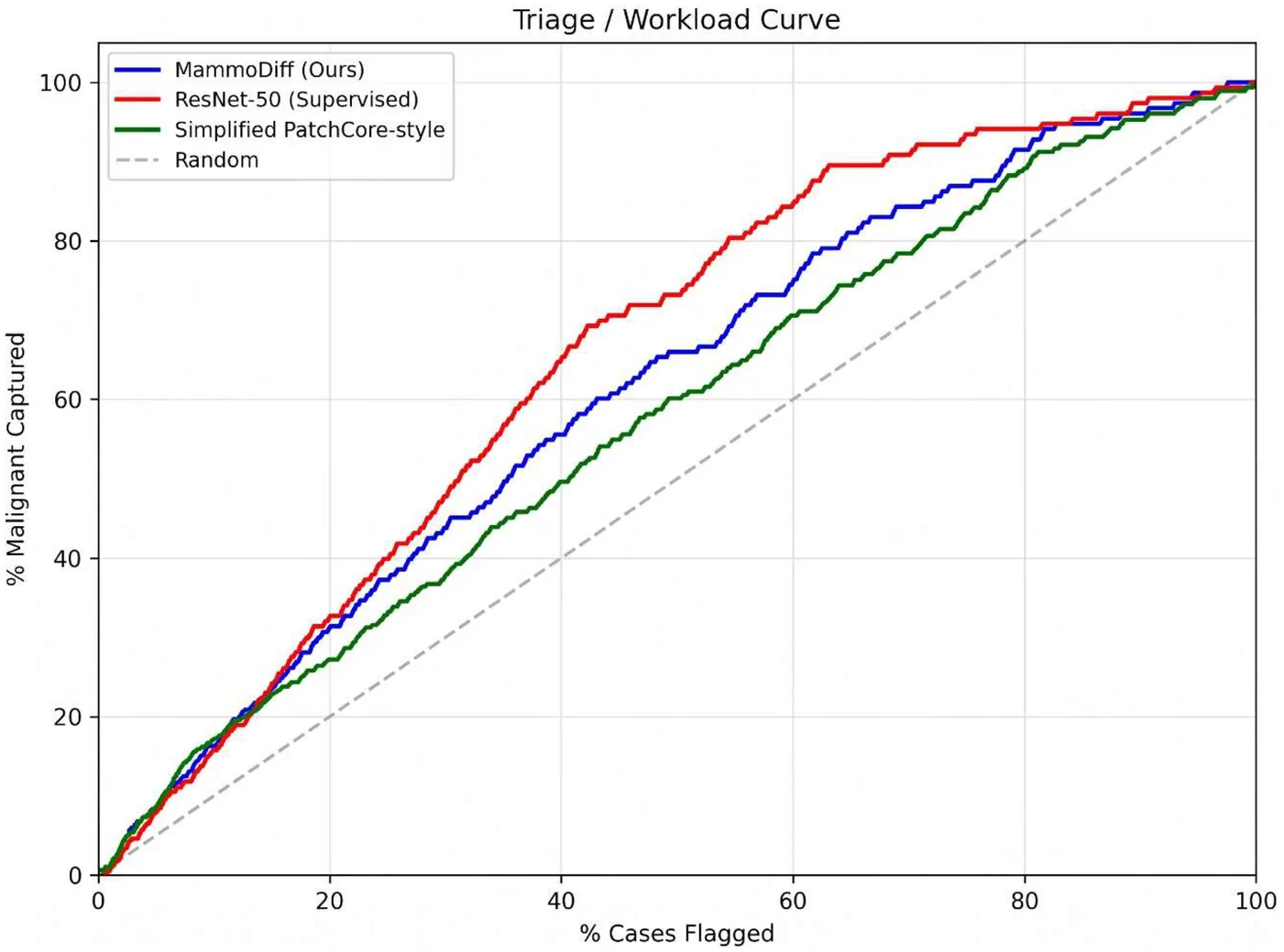
Triage and workload curves for all three methods on the KHCC-mammo test set. The *y*-axis represents the percentage of malignant cases captured when cases are triaged in descending order of anomaly score, and the *x*-axis represents the percentage of the total caseload reviewed. MammoDiff outperforms the simplified PatchCore-style baseline throughout the clinically relevant triage range and approaches the supervised ResNet-50 reference at higher workload fractions.

The clinical operating point analysis in Table 5 reveals an important characteristic of each method. At the high-specificity regime most relevant for screening, where false positive rates must be constrained to prevent excessive recall, MammoDiff achieves sensitivity of 0.471 at 80% fixed specificity, exceeding the simplified PatchCore-style baseline (0.386) by 8.5 percentage points. At 90% specificity, MammoDiff captures 32.7% of malignant cases compared with 26.1% for the simplified PatchCore-style baseline. These results confirm that MammoDiff provides a clinically more useful anomaly ranking than the simplified PatchCore-style feature-distance baseline under the constrained operating conditions that characterise population screening. The supervised ResNet-50 reference achieves higher sensitivity at the 80% and 90% fixed-specificity operating points, with 71.9% sensitivity at 80% specificity and 41.8% at 90% specificity, corresponding to the additional discriminative information conferred by full label supervision.

The triage analysis in Table 6 and Figure 6 provides complementary evidence of MammoDiff's clinical utility. When the top 20% of highest-scoring cases are reviewed, MammoDiff captures 31.4% of all malignant cases in the test set, substantially above the 20.0% expected under random ordering, confirming meaningful case prioritisation. At the top 30% triage threshold, MammoDiff captures 43.8% of malignancies vs. 38.6% for the simplified PatchCore-style baseline, a difference of 5.2 percentage points that is clinically relevant in a screening context. At the very top of the ranked list (top 5% and top 10%), MammoDiff (8.5%, 16.3%) and the simplified PatchCore-style baseline (9.2%, 17.6%) perform comparably, and both are in fact marginally ahead of the supervised ResNet-50 reference (7.8%, 15.7%) in this range; the divergence between MammoDiff and the simplified PatchCore-style baseline widens progressively in the 20%–50% triage range, suggesting that MammoDiff's globally more calibrated anomaly scores are particularly advantageous when a moderate fraction of the caseload is to be reviewed.

**Table 6:** Triage performance: percentage of malignant cases captured when the top k% of highest-scoring cases are flagged for review.

Method	Top 5%	Top 10%	Top 20%	Top 30%
Simplified PatchCore-style	9.2%	17.6%	28.1%	38.6%
MammoDiff (Ours)	8.5%	16.3%	31.4%	43.8%
ResNet-50 (Reference)	7.8%	15.7%	32.7%	47.7%

### 4.6 Internal consistency of the guided translation: healthy-shift index

In addition to image-level discrimination, MammoDiff offers an internal consistency check on the classifier-guided reconstruction by means of the Healthy-Shift Index (HSI) defined as HSI(*x*_0_) = C*φ*(malignant|*x*_0_, *t* = 0) − *C_φ_*(malignant| $x_{0}^{\prime }$, *t* = 0), with a positive HSI value reflecting a successful semantic shift towards the healthy class distribution. Table 7 and Figure 7 show the HSI statistics for all malignant (*n* = 153) and normal (*n* = 151) test cases at the optimal setting (L* = 750, s* = 50).

**Figure 7:**
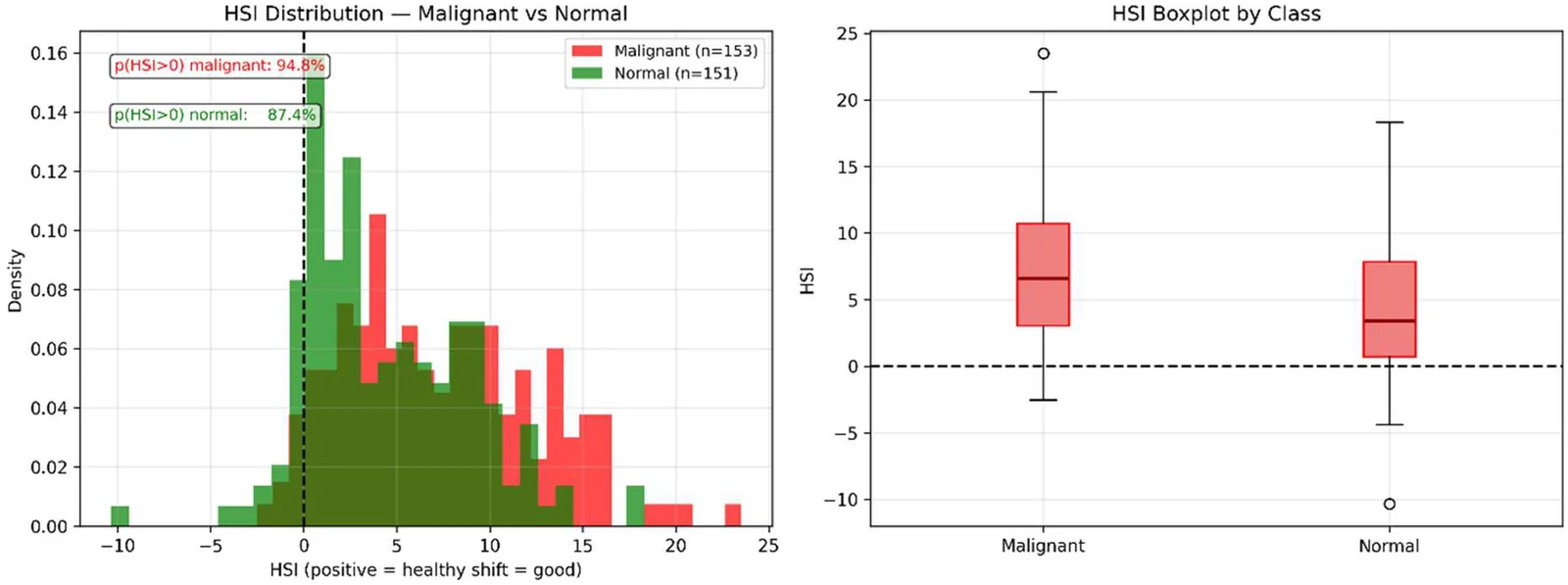
Healthy-Shift index (HSI) distribution for mammoDiff (L* = 750, s* = 50) on the KHCC-mammo test set. Left: density histogram of HSI values for malignant (red, *n* = 153) and normal (green, *n* = 151) cases, with a vertical dashed line at HSI = 0. Right: boxplot comparison by class. Positive HSI values (right of the dashed line) indicate a successful healthy translation. The malignant distribution is substantially right-shifted relative to normal, with *p*(HSI > 0) = 94.8% for malignant cases.

**Table 7:** Healthy-Shift index (HSI) statistics for mammoDiff (L* = 750, s* = 50) on all KHCC-mammo test cases. HSI > 0 indicates successful healthy translation (positive = good). for normal cases, HSI is expected to be near zero as the reconstruction should minimally modify already-healthy anatomy.

Subset	*n*	Mean HSI	Median HSI	p(HSI > 0)
Malignant cases	153	7.327 logit units	6.601 logit units	94.8%
Normal cases (reference)	151	4.369 logit units	3.414 logit units	87.4%

Among the 153 malignant test cases, 94.8% achieve positive HSI, with a mean of 7.327 logit units and a median of 6.601 logit units. However, this figure must be interpreted with care: 87.4% of normal cases also achieve positive HSI, indicating that the classifier gradient applies directional pressure toward the healthy class for nearly all inputs regardless of true pathological status. The clinically meaningful quantity is therefore not *p*(HSI > 0) in isolation, but the distributional gap between classes, the mean HSI for malignant cases (7.327 logit units) exceeds that of normal cases (4.369 logit units) by approximately 2.96 logit units, suggesting that the guidance mechanism applies a proportionally stronger correction to pathological inputs than to healthy ones. We further note that HSI is a self-validation metric: it uses the same noise-conditioned classifier *C_φ_* that drives the guided reconstruction, and therefore measures internal consistency of the pipeline rather than independent clinical confirmation. The 5.2% of malignant cases with negative HSI represent reconstruction failures where the guided decoding did not produce a healthier counterfactual, corresponding predominantly to false-negative classifications in the anomaly score, consistent with the 40.0% false-negative rate at the Youden operating point (FN = 61 of 153).

The results for the normal cases [mean = 4.369, median = 3.414, *p*(HSI > 0) = 87.4%] further underscore this interpretive caution. The non-trivial positive HSI for normal cases means that the classifier guided reconstruction also pushes the normal cases toward slightly lower malignant probability, which is a result of the classifier gradient always being directed towards the healthy class irrespective of the pathological status of the input. This is a normal property of the pipeline, not an anomaly: The guidance mechanism is not conditioned on the true label, and thus the same directional pressure is applied to both normal and malignant inputs. It is the distributional separation that is seen in the data in Figure 7 that is the key diagnostic observation; in the figure, the mean HSI value is 7.327 for the malignant class, while it is 4.369 for the normal class, a difference of 2.958 logit units, which is consistent with the interpretation that the classifier guidance makes larger corrections to pathological inputs than to already healthy ones. This semantic separation is not only orthogonal to the image-level AUROC but also gives a mechanistic validation of the pipeline of counterfactual reconstruction which would not be possible with a discriminative classifier alone.

To characterise this separation quantitatively rather than through the sign of the index alone, we compared the malignant and normal HSI distributions directly. The mean HSI differs by 2.958 logit units and the median by 3.187 logit units, in both cases in favour of the malignant class. Treating the sign of the index as a binary outcome, 145 of 153 malignant cases (94.8%) and 132 of 151 normal cases (87.4%) have a positive HSI, a difference of 7.4 percentage points (Newcombe 95% CI: 0.9–14.1 percentage points; odds ratio 2.61; two-sided Fisher exact *p* = 0.027). The separation is therefore statistically detectable, but it is weak: the lower bound of the interval lies close to zero, and a single index value carries very little information about the class of an individual case. These figures should be read together with the substantial overlap that is visible in Figure 7. The index separates the two classes at the level of the distribution, but the overlap is wide enough that HSI is not usable as a per-case discriminator, and we do not propose it as one.

We would also restate the limit of what HSI can establish. Its role in this work is diagnostic in the engineering sense: it tells us whether the guided reconstruction is moving images in the intended direction within the classifier's semantic space. It cannot tell us whether that movement is clinically correct, because the same classifier both drives the guidance and evaluates the result, so a high HSI is in part a statement about internal consistency. An independent version of the metric would require a second classifier trained separately from the guidance model, preferably with a different architecture and a different initialisation, and evaluated on the same reconstructions. We identify this as necessary work before HSI could be presented as a validation metric rather than as a consistency check, and we have adjusted the wording throughout the manuscript accordingly.

### 4.7 Qualitative analysis: visualization of anomaly maps on the test Set

Besides the quantitative, the qualitative visualization of the anomaly maps created by MammoDiff offers information about the spatial behavior of the classifier-guided diffusion pipeline and its clinical interpretability. Representative results are presented in Figure 8 for malignant and normal cases for the optimal configuration (L* = 750, s* = 50) of the KHCC-Mammo test set. The five panels of each case show the original mammogram x₀, the reconstructed healthy image $x_{0}^{\prime }$, the pixel-wise absolute difference $\left|x_{0}-{x^{\prime }}_{0}\right|$, the anomaly map *a*(*i*,*j*) displayed as a heatmap, and the cumulative classifier gradient saliency map accumulated during guided denoising.

**Figure 8:**
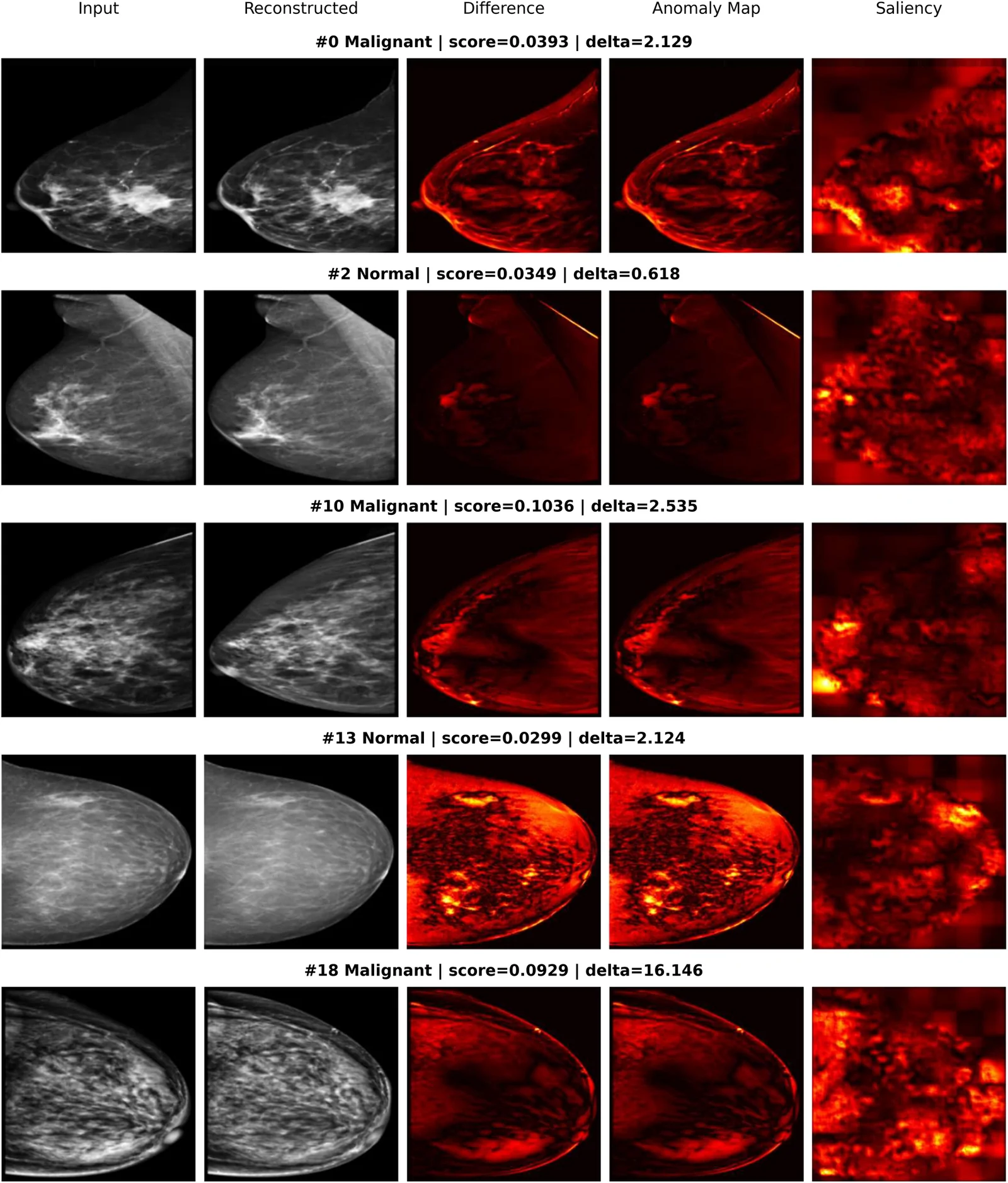
Representative MammoDiff outputs on KHCC-Mammo test cases (*L* = 750, *s* = 50). Each row shows, from left to right, the input x₀, the reconstruction $x_{0}^{\prime }$, the difference map, the anomaly map and the saliency map. Rows 1, 3 and 5 are malignant cases (#0, #10, #18) and rows 2 and 4 are normal cases (#2, #13); each row title gives the image-level anomaly score (score) and the Healthy-Shift Index (delta). Each heatmap is displayed with its own colour scale, so intensities should be compared within a row rather than across rows; the anomaly score provides the image-level comparison. The maps are qualitative visualisations and have not been validated against lesion annotations.

For the malignant cases (rows 1, 3, and 5), the anomaly maps show spatially coherent areas of high intensity, which are indicated by the warm colors in the heatmap and represent the areas where the reconstruction process modified the most according to the classifier. These areas are morphologically similar to clinically suspicious mammographic features such as irregular density foci and regions of locally high glandular contrast, although we do note that, without pixel-to-pixel lesion annotation, we cannot make a formal claim of spatial correspondence with annotated pathological structures. The healthy image $x_{0}^{\prime }$ is reconstructed, and has plausible tissue texture at these locations, suggesting that guidance preferentially modifies areas of pathological appearance, while preserving surrounding anatomy. This selective repainting is a property of the diffusion-based counterfactual reconstruction paradigm, and was not directly compared to GAN and autoencoder baselines in this study.

In the normal cases (rows 2 and 4), the anomaly maps are near-zero throughout the image, confirming that MammoDiff does not introduce spurious anomaly signals when processing genuinely healthy mammograms. The small residual values observed in some normal reconstructions reflect minor stochastic variation accumulated across the DDIM denoising trajectory and remain well below the Youden-optimal classification threshold. This near-zero anomaly pattern in normal cases is consistent with the test-set specificity of 0.7417 reported in 4.3, indicating that the classifier gradient does not systematically disrupt healthy tissue structure. The saliency maps provide a complementary perspective on pipeline interpretability: the spatial regions of highest accumulated classifier gradient activity are qualitatively consistent with the spatial location of elevated anomaly map intensity, suggesting spatial correspondence between the guidance signal and the resulting reconstruction differences. We note, however, that the HSI is a classifier-score shift metric and not a spatial localization metric; confirming the spatial accuracy of the anomaly maps quantitatively requires pixel-level lesion annotations that are not available for the KHCC-Mammo dataset and is identified as future work (Section 5).

The visualization also reveals two recurrent failure modes. First, a fraction of malignant cases produces diffuse, low-intensity anomaly maps that do not reach the binary classification threshold, accounting for the 61 false-negative cases in the confusion matrix. On visual inspection, these cases are predominantly characterized by small, low-contrast lesions presenting against a high-density glandular background, where the classifier gradient appears insufficient to produce a spatially concentrated anomaly signal. A density-stratified quantitative analysis would be required to test this hypothesis formally. ACR density category is recorded at the examination level for this cohort (Figure 1b), but with only 304 test images the resulting density subgroups would contain too few malignant cases for a stable subgroup AUROC estimate, so we did not attempt such an analysis here. It is identified as a priority for the larger multi-centre cohort discussed in Section 5. Second, a small number of cases exhibit boundary artifacts at the pectoral muscle margin and skin-air interface, consistent with the gradient susceptibility at soft-tissue interfaces discussed in 3.7 and the ablation observations in 4.2. Both failure modes are acknowledged as priority targets for the architectural and training improvements identified in Section 5.

To state the status of these visualisations plainly: the anomaly maps presented in this section are qualitative. No pixel-level lesion masks were available for the KHCC-Mammo cohort, no radiologist reader study of localisation accuracy was conducted, and consequently no quantitative localisation metric, whether Dice, pixel-level AUROC, pointing-game accuracy, or lesion-to-background ratio, can be computed from the present data. Where we describe an anomaly map as corresponding to a suspicious region, that description reflects visual inspection by the authors and should be read as illustrative rather than as evidence of localisation performance. We have accordingly avoided the term “localisation” when describing the output of MammoDiff and use “spatial visualisation” throughout. Establishing localisation performance would require either a subset of the cohort annotated with lesion contours by a radiologist, or a reader study in which radiologists score whether the highlighted region corresponds to the reported finding. Both are planned for the extended cohort described in Section 5.

### 4.8 Summary and discussion

MammoDiff achieves AUROC = 0.7246 on a clinically acquired mammographic cohort from King Hussein Cancer Center, Jordan, representing, to our knowledge, the first application of classifier-guided diffusion models to digital mammography. The framework combines three capabilities that we have not seen provided together in the weakly supervised setting: image-level discrimination above the unsupervised baseline evaluated here (AUROC = 0.7246, surpassing the simplified PatchCore-style baseline by 6.1 percentage points), qualitative spatial anomaly visualization through pixel-wise counterfactual reconstruction maps, and an internal consistency check on the pathological-to-healthy translation, with *p*(HSI > 0) = 94.8% across malignant test cases. The supervised ResNet-50, included as a fully labelled reference, achieves AUROC = 0.8100 an 8.5 percentage-point advantage over MammoDiff reflecting the additional discriminative information conferred by full annotation, and should not be interpreted as a performance ceiling.

The hyperparameter ablation identifies *L* = 750, *s* = 50 as the domain-optimal configuration for the KHCC-Mammo dataset, in contrast to the *L* = 500 configuration reported optimal by (18) on BRATS2020 brain MRI and CheXpert chest radiography. We regard this domain shift as a finding that warrants discussion rather than a straightforward advantage: the requirement for deeper noise encoding in mammography likely reflects the greater structural heterogeneity and reduced pathological contrast of mammographic glandular tissue relative to brain MRI, where pathological signal-to-background ratios are typically higher. The deeper encoding also entails substantially longer inference time (∼84 s per image at *L* = 750) compared to the *L* = 500 configuration (∼51 s), a trade-off that would need to be reconsidered for deployment in high-throughput screening workflows.

Several limitations of the present study constrain the interpretation of these findings and are acknowledged explicitly to guide future work. First, the KHCC-Mammo dataset is a single-centre cohort; the generalizability of the L* = 750, s* = 50 configuration and the overall AUROC to other mammographic acquisition protocols, scanner manufacturers, and patient demographics has not been evaluated. Second, the 256 × 256 input resolution substantially attenuates high-frequency microcalcification signal mammographic microcalcifications typically measure 0.1–0.5 mm, spanning only 1–3 pixels at this resolution making detection of clustered microcalcifications, a primary sign of ductal carcinoma *in situ*, essentially impossible at the pixel level. This resolution constraint is expected to contribute to the false-negative rate observed at the Youden operating point. We tested it directly in Section 4.9, where two experiments at an effective resolution of 512 × 512, one applying the present models to patches and one retraining both models on patches, failed to improve discrimination; that analysis indicates that the binding constraint is label granularity rather than resolution, and the limitation is therefore best read jointly with limitation seven below. Third, the anomaly maps are assessed qualitatively only; without pixel-level lesion annotations, quantitative localization metrics such as pointing-game accuracy, lesion-to-background ratio, or pixel-level AUROC cannot be computed. The anomaly map is therefore presented as a qualitative spatial visualization and not as a validated localization method. Fourth, the inference time of approximately 84 s per image at L* = 750 on dual RTX 4090 GPUs would saturate high-end GPU infrastructure at the throughput required for routine mammographic screening (≈100 mammograms per day at KHCC); clinically deployable latency would require either latent-diffusion compression to reduce inference below 10 s, or restriction to a flagged triage subset rather than whole-population screening. Any language suggesting immediate clinical deployability should be interpreted in this context. Fifth, the modest test-set size (*n* = 304) produces wide bootstrap confidence intervals for threshold-dependent metrics, and inter-method differences at the level of 5–8 AUROC percentage points should be interpreted with appropriate caution. Sixth, the HSI is a classifier-score shift metric rather than a spatial localization metric; its high value [*p*(HSI > 0) = 94.8%] reflects internal consistency of the guidance and does not independently validate the spatial accuracy of the anomaly maps. Seventh, and in our present judgement most consequential for the absolute level of performance reported here, the labels are the reporting radiologist's characterisation rather than histopathology, and they are assigned per examination rather than per breast. As set out in Section 3.1, this means that positive examinations may contribute views of an unaffected breast, and that a characterisation resting on documented disease history rather than on a current finding is inherited by images in which no lesion is present. Both factors place a ceiling on any discriminative metric computed on this cohort that is independent of the model, and both are addressable without new imaging: laterality-specific labelling can be derived from fields already present in the source records, and pathology linkage would convert the reference standard from radiological to histological. These limitations notwithstanding, the results establish MammoDiff as a proof-of-concept weakly supervised framework that exceeds the simplified PatchCore-style baseline evaluated here, without our being able to demonstrate that the difference is statistically significant, and that provides interpretability capabilities counterfactual reconstruction and HSI validation that discriminative classifiers cannot offer, laying the groundwork for the extensions and prospective validations identified in Section 5.

Among these limitations we would single out the absence of external validation as the most consequential. Every result in this paper comes from a single institution, a single vendor's FFDM systems, and a single acquisition and reporting protocol, and the hyperparameters L* and s* were selected on a validation set drawn from that same distribution. Published experience with mammographic AI is that performance falls substantially when systems are moved to independent cohorts, and the DREAM Challenge external validation (36) is a direct demonstration of that gap. We would therefore expect the AUROC reported here to be an optimistic estimate of what MammoDiff would achieve on data from another centre, and we would not assume that *L* = 750 and *s* = 50 transfer unchanged. Until an external evaluation is carried out, the results in this paper should be read as characterising the method on KHCC-Mammo, and not as an estimate of its general performance. Table 8 summarises the key findings across all evaluation dimensions.

**Table 8:** Summary of key findings across evaluated methods on the KHCC-mammo test set (*n* = 304).

Dimension	Simplified PatchCore-style	MammoDiff (Ours)	ResNet-50
AUROC	0.6634	0.7246 (+6.1pp)	0.8100
Specificity	0.4967	0.7417	0.8477
Recall	0.7712	0.6013	0.6928
Qualitative spatial visualisation	No	Yes (anomaly map)	No
Annotation required	Normal-only image labels	Image-level binary labels	Image-level binary labels
Internal consistency check	No	Yes [p(HSI > 0) = 94.8%]	No
Inference time/image	∼1 s	∼84 s	∼0.1 s
Supervision level	Normal-only (one-class)	Weakly supervised	Supervised reference

### 4.9 High-resolution sensitivity analysis

Section 3.2.1 argued that the 256 × 256 input resolution should limit the detectability of calcification-scale findings, and Section 4.8 lists this as a limitation of the study. To test whether resolution is in fact the binding constraint, we carried out two further experiments at an effective resolution of 512 × 512.

In both experiments each mammogram is resized to 512 × 512 and divided into a 2 × 2 grid of non-overlapping 256 × 256 patches, so that the model input size is unchanged while the effective resolution is doubled. Each patch is processed independently by the full MammoDiff pipeline, and the four resulting anomaly maps are reassembled into a single 512 × 512 map by Gaussian-weighted averaging (*σ* = 64 px), which suppresses discontinuities at the patch boundaries. Three score-aggregation strategies are evaluated: spatial mean pooling, which matches the main protocol, together with 95th-percentile and maximum pooling, which are more sensitive to spatially concentrated signals.

The two experiments differ only in the models used. In the first, the DDPM and the noise-conditioned classifier trained on whole 256 × 256 images are applied unchanged to the patches. In the second, both models are retrained from scratch on the patches themselves, using identical architecture, batch size, learning rate, schedule and iteration count, so that the effective resolution is the only variable that differs from the main experiment. Each source mammogram yields four patches, so the retrained models see 9,868 training samples drawn from the same 2,467 images and the same patient-level partition; no additional patients or images were introduced. Results for both experiments are given in Table 9.

**Table 9:** Sensitivity analysis at an effective resolution of 512 × 512. Each mammogram is divided into four non-overlapping 256 × 256 patches and the per-patch anomaly maps are reassembled by Gaussian-weighted averaging. “Whole-image models” applies the models of Section 4.3 unchanged; “patch-trained models” retrains the DDPM and the classifier on patches under identical training settings. All values on the held-out KHCC-Mammo test set (*n* = 304); 95% CIs from 1,000 bootstrap resamples.

Configuration	Pooling	AUROC	95% CI	Sensitivity	Specificity	F1
Main: 256 × 256 resize (Section 4.3)	mean	0.7246	[0.6685, 0.7825]	0.6013	0.7417	0.6479
512 × 512 patches, whole-image models	mean	0.6943	[0.6299, 0.7537]	0.5948	0.7115	0.6298
512 × 512 patches, whole-image models	95th pct	0.6692	[0.6072, 0.7288]	0.6275	0.6410	0.6295
512 × 512 patches, whole-image models	maximum	0.5861	[0.5264, 0.6465]	0.8758	0.2949	0.6751
512 × 512 patches, patch-trained models	mean	0.6937	[0.6316, 0.7530]	0.7124	0.5962	0.6708
512 × 512 patches, patch-trained models	95th pct	0.7000	[0.6367, 0.7603]	0.7451	0.5577	0.6786
512 × 512 patches, patch-trained models	maximum	0.6352	[0.5767, 0.6977]	0.5359	0.7051	0.5836

Neither experiment improves discrimination. With the whole-image models applied to patches, mean pooling gives AUROC = 0.6943, which is 3.0 percentage points below the 0.7246 of the main configuration. After retraining on patches the best variant is 95th-percentile pooling at AUROC = 0.7000, still 2.5 points below the main result. All of the confidence intervals in Table 9 overlap substantially with the interval of the main configuration, so these differences are best read as an absence of benefit rather than as a demonstrated degradation. We note in addition that the high-resolution experiments were executed through a separate inference script, so although the same held-out partition was used, the image ordering differs and a paired statistical comparison against the main configuration is not available.

Retraining does, however, change the behaviour of the pooling operator in a way that we regard as informative. With whole-image models applied to patches, 95th-percentile pooling is worse than mean pooling by 2.5 percentage points of AUROC; after retraining on patches it is better by 0.6 points, a swing of roughly three points. A percentile operator can outperform a mean only if the anomaly signal is spatially concentrated, so this reversal indicates that patch training does produce a more localised anomaly map, which is the effect that higher resolution was expected to deliver. The effect is real but small, and it is not large enough to move the image-level AUROC.

The retrained variant also shifts the operating point substantially. Sensitivity rises from 0.6013 in the main configuration to 0.7451 with 95th-percentile pooling, an increase of 14.4 percentage points, while specificity falls from 0.7417 to 0.5577. For a screening application the direction of this trade is the useful one, and we note it because low sensitivity is the principal clinical weakness of the main configuration; but the accompanying loss of specificity is too large for the change to be presented as an improvement.

The explanation we favour is that resolution is not the binding constraint in this setting, and that label granularity is. Under image-level supervision the binary label of a mammogram must be propagated to all four of its patches. For a malignant case the lesion is typically confined to a single patch, so approximately three quarters of the patches carrying a malignant label contain no lesion and are visually indistinguishable from patches labelled normal. The consequence is visible directly in the classifier that supplies the guidance gradient. Averaged over the final 5,000 training iterations, the whole-image classifier reaches 87.4 ± 1.3% accuracy across all timesteps and 99.8 ± 0.1% at low noise levels (*t* < 200), whereas the patch classifier reaches only 65.4 ± 1.3% and 70.3 ± 1.5% respectively, a deficit of 22.0 and 29.5 percentage points. These are training-batch accuracies, which makes the comparison more telling rather than less: the whole-image classifier, with 5.45 M parameters, essentially memorises its training set, while the identical architecture applied to patches drawn from the same images cannot exceed roughly 70%. A model capable of memorisation fails to fit its own training data only when the labels are internally inconsistent, which is the expected signature of the label propagation described above. Because this classifier supplies the gradient that steers the entire reconstruction, its ceiling propagates directly into the quality of the anomaly map.

We therefore conclude that resolution and annotation granularity are coupled in the weakly supervised formulation, and that increasing the former without the latter yields no benefit. Detecting calcification-scale disease reliably will require lesion-level annotation, at least on a subset of the cohort, so that patch labels can be assigned individually rather than inherited from the parent image. We report this negative result in full because it delimits what the present formulation can achieve, and because it identifies the specific resource that would be needed to move beyond it, which is annotation rather than compute or resolution.

### 4.10 Computational feasibility and deployment constraints

Because Section 4.5 discusses triage, it is necessary to be concrete about whether the method could actually run at the volume that a triage application implies. At the selected configuration (L* = 750, s* = 50) a single 256 × 256 image requires approximately 84 s on two RTX 4090 GPUs. Essentially all of that time is spent on the 750 DDIM encoding steps and the 750 guided denoising steps; each step requires a forward pass through the 113.7 M-parameter U-Net, and the guided steps additionally require a backward pass through the classifier to obtain the gradient.

KHCC performs on the order of 100 mammographic examinations on a screening day. A standard bilateral examination yields four images (CC and MLO for each breast), so a full day of work is approximately 400 images. At 84 s per image this amounts to approximately 9.3 h of continuous computation on the two-GPU configuration used here. The method is therefore not impossible to run at the institution's current volume, but it would consume an entire overnight batch window on hardware that is not otherwise idle, it leaves no margin for reprocessing or for growth in volume, and it would fail immediately at the throughput of a national screening programme.

Three routes to a workable latency are available, ordered here by the effort they require. The least invasive is DDIM step striding: the encoding and denoising trajectories can be sub-sampled to 50–100 steps rather than the full L, which prior work reports as feasible with limited loss of sample quality (17, 19), and which would give roughly an order-of-magnitude reduction. The second is latent diffusion (30), which performs the diffusion in a compressed latent space and has been reported to reduce computational cost by a factor of four to eight while maintaining sample quality; this requires retraining but no change to the anomaly-scoring logic. The third, and the one most compatible with the present results, is to abandon whole-population processing and to apply MammoDiff only to a subset already flagged by a fast discriminative model, so that the expensive generative pass is spent only where a spatial visualisation is actually wanted.

We have not implemented any of these, and we therefore do not claim that MammoDiff is deployable in a resource-constrained setting on the grounds of annotation efficiency alone. Annotation efficiency and computational efficiency are separate axes. The present method is favourable on the first and clearly unfavourable on the second, and any claim of practicality has to satisfy both.

## 5 Conclusion and future work

This paper presented MammoDiff, a weakly supervised mammographic anomaly detection framework based on classifier-guided denoising diffusion probabilistic models, applied to, to our knowledge, the first clinically acquired Middle Eastern mammographic cohort evaluated under this paradigm. MammoDiff encodes input anatomy through deterministic DDIM noise encoding and reconstructs a counterfactual pseudo-healthy image via classifier gradient guidance, producing pixel-wise anomaly maps without requiring any pixel-level annotation. The proposed Healthy-Shift Index (HSI) provides an internal consistency check on the guided reconstruction, orthogonal to standard classification metrics; because it uses the same classifier that drives the guidance, it is not an independent validation. On the held-out KHCC-Mammo test set (*n* = 304), MammoDiff achieves AUROC = 0.7246, surpassing the simplified PatchCore-style baseline by 6.1 percentage points, with *p*(HSI > 0) = 94.8% confirming successful healthy translation in the majority of malignant cases. These results position MammoDiff as a proof-of-concept rather than as a deployable system: the sensitivity of 0.6013 at the Youden-optimal threshold, the absence of quantitative localisation validation, the single-centre design without external validation, and the inference time of approximately 84 s per image each preclude clinical use in the present form. What the study does establish is a reproducible and annotation-efficient baseline for weakly supervised mammographic anomaly detection in resource-constrained clinical settings. Future work will focus on external multi-centre validation with pixel-level lesion annotations to enable quantitative localization evaluation, alternative anomaly score aggregation strategies to reduce false negatives, and inference acceleration toward clinically deployable latency.

## Data Availability

The de-identified mammographic images supporting the conclusions of this article, organised by class label, will be made available by the authors upon reasonable request, without undue reservation. The associated radiology reports and clinical metadata cannot be shared, owing to the patient privacy regulations and institutional data governance policies of King Hussein Cancer Center (KHCC), Amman, Jordan. Requests to access the datasets should be directed to Iyad Sultan, isultan@khcc.jo. The source code is publicly available at https://github.com/Ahmadnasayreh/MammoDiff_Final.

## References

[1] Sung H Ferlay J Siegel RL Laversanne M Soerjomataram I Jemal A. Global cancer statistics 2020: gLOBOCAN estimates of incidence and mortality worldwide for 36 cancers in 185 countries. CA Cancer J Clin. (2021) 71:209–49. 10.3322/caac.21660 33538338

[2] Tabár L Dean PB Chen TH-H Yen AM-F Chen SL-S Fann JC-Y. The incidence of fatal breast cancer measures the increased effectiveness of therapy in women participating in mammography screening. Cancer. (2019) 125:515–23. 10.1002/cncr.31840 PMC6588008 30411328

[3] Duffy SW Tabár L Yen AM-F Dean PB Smith RA Jonsson H. Mammography screening reduces rates of advanced and fatal breast cancers. Cancer. (2020) 126:2971–9. 10.1002/cncr.32859 32390151 PMC7318598

[4] Lehman CD Arao RF Sprague BL Lee JM Buist DSM Kerlikowske K. National performance benchmarks for modern screening digital mammography. Radiology. (2017) 283:49–58. 10.1148/radiol.2016161174 27918707 PMC5375631

[5] He K Zhang X Ren S Sun J. Deep residual learning for image recognition. In: CVPR (2016). p. 770–8.

[6] Huang G Liu Z Van Der Maaten L Weinberger KQ. Densely connected convolutional networks. In: CVPR (2017). p. 4700–8.

[7] Dosovitskiy A Beyer L Kolesnikov A Weissenborn D Zhai X Unterthiner T. An image is worth 16×16 words: transformers for image recognition at scale. In: ICLR 2021 (2021).

[8] McKinney SM Sieniek M Godbole V Godwin J Antropova N Ashrafian H. International evaluation of an AI system for breast cancer screening. Nature. (2020) 577:89–94. 10.1038/s41586-019-1799-6 31894144

[9] Zimmerer D Kohl SA Petersen J Isensee F Maier-Hein KH. Context-encoding variational autoencoder for unsupervised anomaly detection. *arXiv* [Preprint]. *arXiv*:1812.05941 (2018).

[10] Chen X Konukoglu E. Unsupervised detection of lesions in brain MRI using constrained adversarial auto-encoders. *arXiv* [Preprint]. *arXiv*:1806.04972 (2018).

[11] Gudovskiy D Ishizaka S Kozuka K. CFLOW-AD: real-time unsupervised anomaly detection with localization via conditional normalizing flows. In: WACV 2022 (2022).

[12] Baumgartner CF Koch LM Tezcan KC Ang JX Konukoglu E. Visual feature attribution using wasserstein GANs. In: CVPR (2018). p. 8309–19.

[13] Siddiquee MMR Zhou Z Tajbakhsh N Feng R Gotway MB Bengio Y. Learning fixed points in generative adversarial networks: from image-to-image translation to disease detection and localization. In: ICCV (2019). p. 191–200. 10.1109/ICCV.2019.00028 PMC7329240 32612486

[14] Meissen F Kaissis G Rueckert D. Challenging current semi-supervised anomaly segmentation methods for brain MRI. *arXiv* [Preprint]. *arXiv*:2109.06023 (2021).

[15] Goodfellow I Pouget-Abadie J Mirza M Xu B Warde-Farley D Ozair S. Generative adversarial nets. NeurIPS. (2014) 27:2672–80. 10.48550/arXiv.1406.2661

[16] Ho J Jain A Abbeel P. Denoising diffusion probabilistic models. NeurIPS. (2020) 33:6840–51. 10.48550/arXiv.2006.11239

[17] Nichol AQ Dhariwal P. Improved denoising diffusion probabilistic models. ICML. (2021) 139:8162–71. 10.48550/arXiv.2102.09672

[18] Wolleb J Bieder F Sandkühler R Cattin PC. Diffusion models for medical anomaly detection. In: MICCAI 2022 (Springer) (2022). p. 35–45.

[19] Song J Meng C Ermon S. Denoising diffusion implicit models. ICLR 2021. *arXiv* [Preprint]. *arXiv*:2010.02502 (2020).

[20] Abdelwahab M Al-Hussaini M Al-Khaldi R. Breast cancer epidemiology in the arab world: a systematic review. Asian Pac J Cancer Prev. (2019) 20:1887–96. 10.31557/APJCP.2019.20.6.1887 31244314 PMC7021628

[21] Shen L Margolies LR Rothstein JH Fluder E McBride R Sieh W. Deep learning to improve breast cancer detection on screening mammography. Sci Rep. (2019) 9:12495. 10.1038/s41598-019-48995-4 31467326 PMC6715802

[22] Nguyen HT Nguyen HQ Pham HH Lam K Le LT Dao M. VinDr-mammo: a large-scale benchmark dataset for computer-aided detection in full-field digital mammography. Sci Data. (2022) 10:277. 10.1038/s41597-023-02100-7 PMC10182079 37173336

[23] Wu N Phang J Park J Shen Y Huang Z Zorin M. Deep neural networks improve radiologists’ performance in breast cancer screening. IEEE Trans Med Imaging. (2020) 39:1184–94. 10.1109/TMI.2019.2945514 31603772 PMC7427471

[24] Baur C Denner S Wiestler B Navab N Albarqouni S. Autoencoders for unsupervised anomaly segmentation in brain MR images: a comparative study. Med Image Anal. (2021) 69:101952. 10.1016/j.media.2020.101952 33454602

[25] Wolleb J Sandkühler R Cattin PC. DescarGAN: disease-specific anomaly detection with weak supervision. In: MICCAI 2020 (Springer) (2020). p. 14–24.

[26] Rudolph M Wandt B Rosenhahn B. Same same but DifferNet: semi-supervised defect detection with normalizing flows. In: WACV 2021 (2021).

[27] Roth K Pemula L Zepeda J Schölkopf B Brox T Gehler P. Towards total recall in industrial anomaly detection (PatchCore). In: CVPR (2022). p. 14318–28.

[28] Dhariwal P Nichol A. Diffusion models beat GANs on image synthesis. NeurIPS. (2021) 34:8780–94. 10.48550/arXiv.2105.05233

[29] Ho J Salimans T. Classifier-free diffusion guidance. NeurIPS 2021 Workshop on Deep Generative Models. *arXiv* [Preprint]. *arXiv*:2207.12598 (2022).

[30] Rombach R Blattmann A Lorenz D Esser P Ommer B. High-resolution image synthesis with latent diffusion models. In: CVPR (2022). p. 10684–95.

[31] Wyatt J Leach A Schmon SM Willcocks CG. AnoDDPM: anomaly detection with denoising diffusion probabilistic models using simplex noise. In: CVPRW (2022). p. 650–6.

[32] Bercea CI Wiestler B Rueckert D Albarqouni S. Generalizing unsupervised anomaly detection: towards unbiased pathology screening. In: MIDL 2023, PMLR (2023).

[33] Pinaya WHL Graham MS Gray R Da Costa PF Tudosiu P-D Wright P. Fast unsupervised brain anomaly detection and segmentation with diffusion models. In: MICCAI 2022 (2022). p. 705–14. 10.1007/978-3-031-16452-1_67

[34] Kim H-E Kim HH Han B-K Kim KH Han K Nam H. Changes in cancer detection and false-positive recall in mammography using artificial intelligence: a retrospective, multireader study. Lancet Digit Health. (2020) 2:e138–48. 10.1016/S2589-7500(20)30003-0 33334578

[35] Sun L Wang J Hu Z Xu Y Cui Z. Multi-view convolutional neural networks for mammographic image classification. IEEE Access. (2019) 7:126273–82. 10.1109/ACCESS.2019.2939167

[36] Schaffter T Buist DSM Lee CI Nikulin Y Ribli D Guan Y. Evaluation of combined artificial intelligence and radiologist assessment to interpret screening mammograms. JAMA Netw Open. (2020) 3:e200265. 10.1001/jamanetworkopen.2020.0265 32119094 PMC7052735

[37] D'Orsi CJ Sickles EA Mendelson EB Morris EA. ACR BI-RADS® Atlas, Breast Imaging Reporting and Data System. 5th ed. Reston, VA: American College of Radiology (2013).

[38] Vaswani A Shazeer N Parmar N Uszkoreit J Jones L Gomez AN. Attention is all you need. NeurIPS. (2017) 30:5998–6008. 10.48550/arXiv.1706.03762

[39] Youden WJ. Index for rating diagnostic tests. Cancer. (1950) 3:32–5. 10.1002/1097-0142(1950)3:1<32::AID-CNCR2820030106>3.0.CO;2-3 15405679 10.1002/1097-0142(1950)3:1<32::aid-cncr2820030106>3.0.co;2-3

[40] Butt AUR Asif M Rashid T Ali H Raoof S Raoof F. Beyond boundaries: a novel ensemble approach for breast cancer detection in ultrasound imaging using deep learning. In: 2024 21st International Bhurban Conference on Applied Sciences and Technology (IBCAST) (IEEE) (2024). p. 315–20.

[41] Khan I Siddique MZ Butt AUR Mudassir AI Qadir MA Munir S. Towards skin cancer classification using machine learning and deep learning algorithms: a comparison. Int J Innov Sci Technol. (2021) 3:110–8. 10.33411/IJIST/2021030508

[42] Ashraf R Kiran I Mahmood T Butt AUR Razzaq N Farooq Z. An efficient technique for skin cancer classification using deep learning. In: 2020 IEEE 23rd International Multitopic Conference (INMIC) (IEEE) (2020),. p. 1–5.

[43] Butt AUR Hamid I Nawaz Q Mahmood T Zhang X Yaqub M. A novel multi-scale deep learning approach for the early detection of Alzheimer’s disease using fMRI. In: 2024 5th International Conference on Computer, Big Data and Artificial Intelligence (ICCBD+AI) (IEEE) (2024). p. 85–90.

[44] Rehman MU Naseem S Butt AUR Mahmood T Khan AR Khan I. Predicting coronary heart disease with advanced machine learning classifiers for improved cardiovascular risk assessment. Sci Rep. (2025) 15:13361. 10.1038/s41598-025-96437-1 40247042 PMC12006408

[45] Khan FA Butt AUR Asif M Aljuaid H Adnan A Shaheen S. Burnt human skin segmentation and depth classification using deep convolutional neural network (DCNN). J Med Imaging Health Inform. (2020) 10:2421–9. 10.1166/jmihi.2020.3258

[46] Ur Rehman A Asif M Qasim KU Ishak MK Khan I Khan J. AdvancedHybridNet: an AI-powered hybrid ensemble for high-accuracy thyroid disease diagnosis using dynamic feature selection (2025).

