# Supplementary material for: MammoDiff: weakly supervised mammographic anomaly detection via classifier-guided denoising diffusion models—a real-world clinical study on the KHCC mammography cohort
Source: Front Med Technol. 2026 Oct 12;8:1912196. doi: 10.3389/fmedt.2026.1912196 (PMC13696467; doi:10.3389/fmedt.2026.1912196)
Supplement: Supplementary file 1 [file Table1.docx]

**Appendix A.  MammoDiff V2, Cosine Schedule with Classifier-Free Guidance**

This appendix reports the complete experimental results for the MammoDiff V2 variant, which incorporates the cosine noise schedule (Nichol & Dhariwal 2021) and class-conditional training with classifier-free guidance (Ho & Salimans 2022). V2 results are presented separately from the main paper because its performance on the KHCC-Mammo test set (n=304) falls below that of the V1 linear-schedule variant reported in Section 4, with a best AUROC of 0.5933 compared to V1's 0.7246. A diagnostic analysis of the performance gap and its likely causes is provided in A.4.

**A.1 Model Configuration**

The V2 DDPM backbone shares the same U-Net architecture as V1 (128 channels, channel multipliers (1,1,2,2,4,4), attention at 16×16, 113,670,786 parameters) but was trained with the cosine noise schedule (Eq. 7–8) and class-conditional label dropout (p_uncond = 0.1) for classifier-free guidance. The noise-conditioned classifier (5,452,098 parameters) was trained independently with the same mixed-timestep sampling strategy as V1. The hyperparameter ablation follows the identical 3×3 grid protocol: L ∈ {250, 500, 750}, s ∈ {50, 100, 200}, evaluated on the held-out KHCC-Mammo test set (n=304; 151 normal, 153 malignant).

Classifier-free guidance (Ho & Salimans 2022) was applied during V2 training by randomly dropping the class condition with probability $p_{uncond}$ = 0.1, training the model jointly on conditioned and unconditional objectives. At inference, the conditional and unconditional score estimates are combined via:

${\varepsilon̂}_{\theta}(xₜ, t, c) =\left( 1+w \right)\varepsilon_{\theta}\left( x_{t},t,c \right)-w\varepsilon_{\theta}\left( x_{t},t,\emptyset\right)$

where w is the guidance weight and *∅* denotes the null class token. In the current experiments, *w* was set to 0 during inference (unconditional guidance disabled), as guidance weight tuning was not performed within the present study timeline.

**A.2 Hyperparameter Ablation, V2**

**Table A1***. Ablation grid results for MammoDiff V2 (cosine schedule + CFG) on the KHCC-Mammo test set (n=304). Best AUROC per noise level highlighted. All AUROC values are near the random-chance baseline of 0.5.*

| **L** | **s** | **AUROC** | **Accuracy** | **F1** | **Δ vs V1** | **Time (min)** |
| --- | --- | --- | --- | --- | --- | --- |
| 250 | 50 | 0.5642 | 0.6117 | 0.4915 | −0.1372 | 98.2 |
| 250 | 100 | 0.5721 | 0.6117 | 0.5000 | −0.1295 | 99.3 |
| 250 | 200 | 0.5793 | 0.6278 | 0.5106 | −0.1234 | 99.1 |
| 500 | 50 | 0.5833 | 0.6084 | 0.5217 | −0.1304 | 201.3 |
| 500 | 100 | 0.5869 | 0.6149 | 0.5370 | −0.1264 | 201.4 |
| 500 | 200 | 0.5896 | 0.6117 | 0.5420 | −0.1247 | 200.0 |
| 750 | 50 | 0.5917 | 0.5825 | 0.5981 | −0.1354 | 301.8 |
| 750 | 100 | 0.5923 | 0.5825 | 0.6079 | −0.1302 | 300.8 |
| **750** | **200** | **0.5933** | 0.5761 | 0.6135 | −0.1338 | 294.8 |

Three observations stand out from Table A1. First, AUROC across all nine configurations falls within the narrow band [0.5642, 0.5933] all near the random-chance baseline of 0.5 and substantially below any V1 configuration. Second, unlike V1 where increasing L consistently improved AUROC and lower s was preferred at L=750, V2 shows a monotonically increasing relationship between gradient scale s and AUROC at every noise level, suggesting that the V2 classifier provides weaker directional guidance and requires larger s values to generate a meaningful anomaly signal. Third, the inference time for V2 is marginally shorter than V1 at all noise levels (e.g., 294.8 min vs 319.0 min at L=750) due to slightly fewer denoising function evaluations under the cosine schedule, but this computational advantage is not clinically meaningful given the performance deficit.

**A.3 Final Evaluation Results, V2 (L=750, s=200)**

**Table A2.** *Final evaluation metrics for MammoDiff V2 at the ablation-optimal configuration (L=750, s=200) on the KHCC-Mammo test set (n=304), compared to V1 and baselines.*

| **Method** | **AUROC** | **Accuracy** | **F1** | **Precision** | **Recall** | **Specificity** | **Supervised** |
| --- | --- | --- | --- | --- | --- | --- | --- |
| PatchCore (Unsup.) | 0.6634 | 0.6343 | 0.6762 | 0.6020 | 0.7712 | 0.5000 | No |
| **MammoDiff V2 (Ours)** | 0.5933 | 0.5761 | 0.6136 | 0.5591 | 0.6797 | 0.4744 | No |
| **MammoDiff V1 (Ours)** | **0.7246** | 0.6711 | 0.6479 | 0.7023 | 0.6013 | 0.7417 | No |
| ResNet-50 (Supervised) | 0.7972 | 0.7378 | 0.7340 | 0.7491 | 0.7190 | 0.7500 | Yes |

**Figure A1.** *ROC curve for MammoDiff V2 (L=750, s=200) on the KHCC-Mammo test set. AUROC = 0.5933. The curve hugs the diagonal, indicating near-random discriminative performance for V2 under current training conditions.*

The best performance of MammoDiff V2 is AUROC = 0.5933, which is significantly lower than that of V1 (0.7246, −13.1 pp) and the unsupervised PatchCore baseline (0.6634, −7.0 pp). The confusion matrix shows that there are 104 true positives, 82 false positives, 74 true negatives and 49 false negatives, which gives a specificity of only 0.4744, which is lower than the random-chance level of 0.5. This means that, on average, anomaly scores are higher for normal cases than for some malignant cases, indicating a systematic bias in the distribution of anomaly maps under the cosine-schedule DDPM. The recall value of 0.6797 is significantly higher than V1's 0.6013, which means that in absolute terms V2 has a higher number of high anomaly cases, but also has a higher number of false-positive cases.

***A.3.1 Healthy-Shift Index , V2***

**Table A3.** *Healthy-Shift Index statistics for MammoDiff V2 on the 153 malignant KHCC-Mammo test cases.*

| **Subset** | **Mean HSI** | **Median HSI** | **p(HSI>0)** |
| --- | --- | --- | --- |
| **Malignant cases (n=153)** | 7.454 logit units | 6.202 logit units | **92.16%** |
| V1 reference | 7.327 | 6.601 | 94.8% |

The HSI analysis shows that there is a significant finding, even though the AUROC is low, 92.16% of V2 malignant cases still get a positive HSI (compared to 94.8% for V1) and the average HSI for V2 is 7.454 logit units vs 7.327 logit units for V1. This validates that the V2 classifier-guided reconstruction is working as intended at the semantic level, in that it is translating the majority of cases with high malignant probability towards lower malignant probability. The performance gap is thus observed as an image-level anomaly score A(x₀) = mean(a(i,j)), indicating that V2 is reconstructing the image with a broader but smaller magnitude of anomaly signal in non-pathological areas compared to V1, which shows a higher magnitude of anomaly signal in pathological areas. This diffuse anomaly pattern reduces the average score of the malignant samples and increases the average score of the normal samples, thus shrinking the score distributions and worsening the AUROC.

**A.4 Diagnostic Analysis of V2 Underperformance**

The substantial performance gap between V1 (AUROC=0.7246) and V2 (AUROC=0.5933) is not a reflection of the cosine schedule's theoretical inferiority which is well-established to be superior for preserving low-noise structural information (Nichol & Dhariwal 2021), but rather the result of training pipeline misalignment specific to the current KHCC-Mammo experiment. Three factors are identified as the primary causes:

***A.4.1 Classifier–DDPM Schedule Mismatch***

The most likely primary cause is that the noise-conditioned classifier Cφ used for V2 inference was trained using the linear noise schedule (V1), as the V2-specific cosine-schedule classifier had not completed its full 60,000-iteration training cycle within the experimental timeline. The classifier gradient ∇_{xₜ} log Cφ(h|xₜ, t) is therefore calibrated to the noise level distribution of the linear schedule, but at inference it receives noisy images xₜ drawn from the cosine-schedule DDPM trajectory a distributional mismatch that systematically biases the gradient magnitude and direction at every denoising step. Under the cosine schedule, the signal-to-noise ratio at low t is substantially higher than under the linear schedule; the linear-schedule classifier therefore underestimates the healthy-class gradient at low-t steps where the cosine DDPM has already preserved significant structure, resulting in under-guidance in the semantically meaningful noise regime.

***A.4.2 Classifier-Free Guidance Weight Untuned***

The V2 DDPM was trained with classifier-free guidance, but the inference guidance weight w (Eq. 11) was set to w=0 for the anomaly detection pipeline, equivalent to using the conditional score only rather than being tuned as an additional hyperparameter. An ablation over w ∈ {0, 1, 2, 3} at the optimal (L, s) would be expected to improve AUROC by reducing the diffuse background reconstruction variation that characterizes the V2 anomaly maps.

***A.4.3 Insufficient Training***

The V2 DDPM cosine-schedule model, while converged according to training loss monitoring, may not have reached the same generative quality as the V1 model trained for 80,000 iterations. Cosine-schedule DDPMs are known to require more careful learning rate scheduling and potentially more training iterations to reach equivalent sample quality to linear-schedule models (Nichol & Dhariwal 2021), particularly for complex texture distributions such as mammographic glandular tissue.

**A.5 Direct Comparison: V1 vs. V2**

**Table A4***. Side-by-side comparison of V1 (linear schedule) and V2 (cosine schedule + CFG) at their respective optimal configurations on the KHCC-Mammo test set.*

| **Configuration** | **AUROC** | **Accuracy** | **F1** | **Recall** | **Specificity** | **HSI p>0** |
| --- | --- | --- | --- | --- | --- | --- |
| **V1: Linear, L=750, s=50** | **0.7246** | 0.6711 | 0.6479 | 0.6013 | **0.7417** | 94.8% |
| **V2: Cosine+CFG, L=750, s=200** | 0.5933 | 0.5761 | 0.6136 | 0.6797 | 0.4744 | 92.16% |
| **Δ (V2 − V1)** | −0.1313 | −0.0950 | −0.0343 | +0.0784 | −0.2673 | −2.61 pp |

As shown in Table A4, the comparison of V2 with V1 validates that V2 is worse than V1 in all the primary metrics except recall (+7.8 pp) and F1 (+3.2 pp). Most importantly, V2's specificity of 0.4744 is 26.7 percentage points lower than V1 and lower than the point of random chance a clinically unacceptable operating point for a screening application. The high recall and catastrophic specificity are typical of a threshold bias problem: the V2 anomaly score is systematically higher in normal cases and the Youden-optimal threshold is set so low that almost all cases (malignant and normal) are classified as positive. The HSI data for V2 (92.16% positive shift) also indicates that this is not a fundamental problem with the generative pipeline, but rather a problem with the distribution of anomaly scores that could be corrected with the remedies outlined in A.4.

**A.6 Planned Corrections for V2**

The following targeted corrections are planned to bring V2 to competitive performance in follow-up work based on the diagnostic analysis in A.4:

- Train a dedicated cosine-schedule noise-conditioned classifier with the V2 DDPM with schedule alignment between the generative model and the guidance signal.
- Run an ablation to determine the best balance between conditional and unconditional score estimates, by varying the guidance weight w in the ablation set as {0.5, 1.0, 2.0, 3.0}.
- Train V2 DDPM for 100,000 iterations with cosine-annealing warm restarts to get full convergence of the cosine-schedule generative model.
- Test alternatives to score aggregation for V2 anomaly maps (95th percentile intensity, maximum patch score) to mitigate the AUROC deficit, which was found to be due to the diffuse background reconstruction artefacts.

Preliminary analysis suggests that the corrected V2 pipeline has the potential to surpass V1, as the theoretical advantages of the cosine schedule, superior structural preservation at low noise levels, more gradual boundary transitions, and better microcalcification texture retention, remain intact as evidenced by the competitive HSI statistics. The present V2 results are reported transparently in this appendix to provide a complete experimental record and to document the baseline against which the corrected V2 will be compared in future work.
